# Comparative Efficacy of Tirzepatide Versus Semaglutide for Weight Loss in Adults With Overweight or Obesity: A Systematic Review and Meta‐Analysis of Head‐to‐Head Studies

**DOI:** 10.1111/cob.70111

**Published:** 2026-08-30

**Authors:** Giovanna Pagani Paccola, Roberta Fernandes de Oliveira, Matheus Menão Mochetti, Fernanda Pataro Marsola Razera, Rafael Vecchi, Renan Canale Peres Montanher

**Affiliations:** ^1^ Faculdade de Medicina de Jaú Universidade do Oeste Paulista Jaú São Paulo Brazil; ^2^ Faculdade de Medicina de Bauru Universidade de São Paulo Bauru São Paulo Brazil

**Keywords:** obesity, semaglutide, tirzepatide, weight loss

## Abstract

This systematic review and meta‐analysis aimed to compare the efficacy and safety of tirzepatide versus semaglutide for weight reduction in adults with overweight or obesity. We included randomised controlled trials and observational studies comparing tirzepatide and semaglutide with ≥ 24 weeks of follow‐up. The primary outcome was percentage weight change from baseline. Secondary outcomes included absolute weight change, weight‐loss thresholds, HbA1c and safety outcomes. Ten studies including 41 381 participants were analysed. Tirzepatide was associated with greater percentage weight reduction than semaglutide (MD −4.28 percentage points; 95% CI −5.28 to −3.28; *p* < 0.00001) and greater absolute weight loss (MD −4.43 kg; 95% CI −5.56 to −3.30; *p* < 0.00001). Tirzepatide was also associated with a higher likelihood of achieving ≥ 10%, ≥ 15% and ≥ 20% weight loss, with no difference at ≥ 5%. HbA1c reduction was greater with tirzepatide (MD −0.29%; *p* = 0.0002). Subgroup analyses by study design and type 2 diabetes status yielded consistent findings. There was no significant difference in treatment discontinuation due to adverse events (RR 1.28; *p* = 0.54), whereas serious adverse events were more frequent with tirzepatide (RR 1.83; *p* = 0.007). Overall and gastrointestinal adverse events were similar between groups. Tirzepatide was associated with greater weight reduction, greater glycaemic benefit and a higher likelihood of achieving weight‐loss thresholds than semaglutide, but with a higher risk of serious adverse events.

## Highlights

- What is already known about this subject?
  ○ Semaglutide and Tirzepatide are potent pharmacological therapies for obesity, but direct head‐to‐head comparative evidence on their effectiveness and safety remains limited.
  ○ Previous comparative meta‐analyses have generally reported greater weight loss with tirzepatide, but many relied on indirect comparisons or included heterogeneous populations and broader eligibility criteria, limiting clinical comparability.
  ○ Although categorical weight‐loss thresholds have been evaluated in prior meta‐analyses, uncertainty remains regarding the consistency and clinical applicability of these findings across clinically comparable patient populations.
- What this study adds?
  ○ This study provides one of the most comprehensive head‐to‐head meta‐analyses to date, including more than 41 000 participants and integrating randomised controlled trials and real‐world observational evidence.
  ○ By applying stricter eligibility criteria and excluding clinically distinct populations (e.g., prior bariatric surgery and type 1 diabetes), this study improves comparability and clinical generalisability.
  ○ By requiring at least 24 weeks of follow‐up and evaluating categorical weight‐loss targets up to ≥ 20%, this study provides a more clinically relevant assessment of comparative effectiveness.
  ○ The findings highlight an important clinical trade‐off: tirzepatide was associated with greater weight reduction but also a higher risk of serious adverse events than semaglutide.

## 1 Introduction

Obesity is a chronic, relapsing, multifactorial disease characterised by excess adiposity and substantial morbidity and mortality. According to the World Health Organization, in 2022 approximately 2.5 billion adults worldwide were overweight, of whom more than 890 million were living with obesity, representing nearly one in eight individuals globally [1]. More recent global estimates extending through 2024 indicate substantial regional variation, with adult obesity prevalence stabilising in several high‐income countries but continuing to rise or accelerate in most low‐ and middle‐income countries [2]. Overweight and obesity are defined according to body mass index (BMI), with thresholds of BMI ≥ 25 and ≥ 30 kg/m^2^, respectively. Obesity remains a major global health challenge and is associated with a markedly increased risk of metabolic, cardiovascular, musculoskeletal, neurodegenerative and psychiatric disorders, as well as several types of cancer. Furthermore, obesity is linked to impaired quality of life and increased all‐cause mortality [3, 4].

Given its scale and clinical consequences, the effective long‐term management of obesity has become a major public health priority. Robust evidence indicates that sustained weight loss of ≥ 10% is associated with clinically meaningful improvements in obesity‐related health outcomes, including reductions in cardiometabolic risk and systemic inflammation [5, 6]. However, lifestyle‐based interventions, including dietary modification and increased physical activity, are often limited by poor long‐term adherence and weight‐loss maintenance. Consequently, pharmacological therapies have increasingly been incorporated as adjunctive treatments for obesity [6].

Glucagon‐like peptide‐1 receptor agonists (GLP‐1 RAs), initially developed for the treatment of type 2 diabetes mellitus, have emerged as highly effective agents for weight management in individuals both with and without diabetes [7]. These medications mimic the incretin hormone GLP‐1, released by enteroendocrine L cells in response to nutrient ingestion. Activation of GLP‐1 receptors promotes satiety, suppresses appetite, reduces caloric intake, delays gastric emptying and improves glucose homeostasis [8, 9]. Semaglutide, a long‐acting GLP‐1 receptor agonist, has become an established therapy for chronic weight management in patients who do not achieve adequate weight reduction with lifestyle interventions alone.

Glucose‐dependent insulinotropic polypeptide (GIP) is another incretin hormone secreted by K cells that plays a complementary role in metabolic regulation. GIP enhances glucose‐dependent insulin secretion and stimulates glucagon release during euglycemia or hypoglycaemia [10]. Beyond its glycaemic effects, emerging evidence suggests that GIP signalling may also influence central appetite regulation and adipose tissue metabolism, contributing to reduced energy intake, modulation of lipid storage and attenuation of ectopic fat deposition [9, 11].

Tirzepatide, a dual GIP and GLP‐1 receptor agonist initially developed for the treatment of type 2 diabetes, has shown greater weight‐loss efficacy than semaglutide [12, 13]. In SURMOUNT‐5, the estimated mean body weight reduction at 72 weeks was 20.2% with tirzepatide and 13.7% with semaglutide among adults with overweight or obesity and without diabetes [14]. This difference may partly reflect complementary GIP and GLP‐1 receptor activation [9, 11].

Although direct head‐to‐head trials such as SURMOUNT‐5 have demonstrated greater weight loss with tirzepatide than semaglutide, such comparative studies remain limited in number. In addition, heterogeneity in study design, patient populations and reported effect sizes complicates interpretation of the available data. A rigorous synthesis is therefore needed to compare their relative efficacy. Recent systematic reviews have evaluated head‐to‐head comparisons between tirzepatide and semaglutide; however, several additional studies have since been published, warranting an updated synthesis of the most recent evidence [15].

Accordingly, this systematic review and meta‐analysis aimed to directly compare the effects of tirzepatide and semaglutide on body weight reduction in adults with overweight or obesity, to inform evidence‐based clinical decision‐making and guide future research priorities. Building upon prior evidence, this study focuses exclusively on direct head‐to‐head comparisons, incorporates both randomised and real‐world data, and applies stricter eligibility criteria to enhance clinical comparability and interpretability.

## 2 Materials and Methods

### 2.1 Study Design

This systematic review and meta‐analysis was conducted in accordance with the methodological standards of the Cochrane Collaboration and is reported in compliance with the PRISMA Statement (Preferred Reporting Items for Systematic Reviews and Meta‐Analyses) [16]. The study protocol was prospectively registered in PROSPERO (International Prospective Register of Systematic Reviews; registration number CRD420261292090).

### 2.2 Search Strategy

A comprehensive search of PubMed, Scopus, the Cochrane Central Register of Controlled Trials and Web of Science was conducted from database inception to 23 February 2026, without restrictions on publication year. Only studies published in English or with an available English translation were considered. The search strategy combined controlled vocabulary and free‐text terms related to tirzepatide and semaglutide. Reference lists of included studies and relevant systematic reviews were also screened for additional eligible studies. The full search strategy is provided in Supporting Information 1.

### 2.3 Eligibility Criteria

Randomised controlled trials (RCTs) and observational cohort studies directly comparing subcutaneous tirzepatide and subcutaneous semaglutide at any dose regimen evaluated in the original study were eligible. Across the included studies, doses ranged from 2.5 to 15 mg once weekly for tirzepatide and from 0.25 to 2.4 mg once weekly for semaglutide. Studies were required to include adults (≥ 18 years) with overweight or obesity (BMI ≥ 25 kg/m^2^) and a minimum follow‐up of 24 weeks.

Eligibility criteria followed the PICOS framework: population (adults with overweight or obesity), intervention (tirzepatide), comparator (semaglutide), outcomes (primary outcome: percentage change in body weight from baseline; secondary outcomes: absolute body weight change in kilograms, glycaemic control [HbA1c] and safety outcomes including treatment discontinuation due to adverse events and serious adverse events when consistently reported), and study design (RCTs or observational cohort studies).

Studies were excluded if they compared either medication with placebo or other pharmacologic agents, had follow‐up shorter than 24 weeks, were conducted in animal models or did not report weight change outcomes in a form suitable for quantitative synthesis. Studies that did not clearly define the study population according to BMI criteria for overweight or obesity were also excluded. Case reports, narrative reviews, opinion articles, cross‐sectional studies and conference abstracts without full peer‐reviewed manuscripts were excluded. Studies conducted exclusively in specific populations (e.g., type 1 diabetes, prior bariatric surgery or active malignancy) were also excluded. When multiple reports were based on the same or substantially overlapping study population, as determined by the data source, study setting, recruitment period, eligibility criteria and participant characteristics, only one report was included to avoid double‐counting. Preference was given to the report with the largest sample size or, when sample sizes were similar, the report providing the most complete outcome data and longest follow‐up.

### 2.4 Study Selection and Data Extraction

Study selection was performed independently by two reviewers in two stages: initial screening of titles and abstracts followed by full‐text review of potentially eligible studies. Discrepancies were resolved by discussion and consensus, with consultation of a third reviewer when necessary. The selection process was documented using a PRISMA flow diagram.

Data extraction was performed independently using a standardised, prespecified form. Extracted variables included study characteristics (first author, year, country, design), sample size, follow‐up duration, dosing regimen, outcome assessment methods and reported efficacy and safety outcomes. Adjusted and unadjusted effect estimates were extracted separately when available. For quantitative synthesis, we used arm‐level aggregate outcome estimates, including reported group means, least‐squares means or arm‐level estimates from matched analytic populations, with corresponding variability measures for continuous outcomes and event counts with total sample sizes for dichotomous outcomes. Adjusted between‐group regression estimates, hazard ratios and odds ratios were not pooled together with unadjusted aggregate‐data estimates. Only data reported in published manuscripts and Supporting Information were included.

### 2.5 Risk‐of‐Bias Assessment

Risk‐of‐bias assessments were performed independently by two reviewers. Disagreements were resolved through discussion and consensus. RCTs were assessed using the Cochrane Risk of Bias 2 (RoB 2) tool, and nonrandomised observational cohort studies were evaluated using the Risk of Bias in Non‐randomised Studies of Interventions (ROBINS‐I) tool [17, 18]. Each study was categorised according to the respective instrument's criteria.

### 2.6 Statistical Analysis

The primary outcome was the mean percentage change in body weight from baseline. Secondary outcomes included absolute weight change (kg), HbA1c and safety outcomes when sufficient data were available. For the overall pooled analyses, when multiple follow‐up time points were reported, data from the longest available follow‐up were used.

Given anticipated clinical and methodological heterogeneity, pooled estimates were calculated using a random‐effects model. Continuous outcomes were expressed as mean differences (MDs) and dichotomous outcomes as risk ratios (RRs), both with 95% confidence intervals (CIs). Statistical heterogeneity was assessed using Cochran's *Q* test and quantified with the *I* ^2^ statistic. Prespecified subgroup and sensitivity analyses were performed to explore sources of heterogeneity and evaluate the robustness of the findings. Publication bias and small‐study effects were assessed for the primary outcome by visual inspection of a funnel plot and Egger's regression test, as 10 studies contributed to this analysis. Additional details regarding statistical procedures are provided in Supporting Information 2.

All statistical tests were two‐sided, with *p* < 0.05 considered statistically significant. Analyses were conducted using Review Manager version 5.4 and jamovi version 2.6.44 with the MAJOR meta‐analysis module for publication bias analyses.

## 3 Results

### 3.1 Study Selection and Characteristics

**FIGURE 1:**
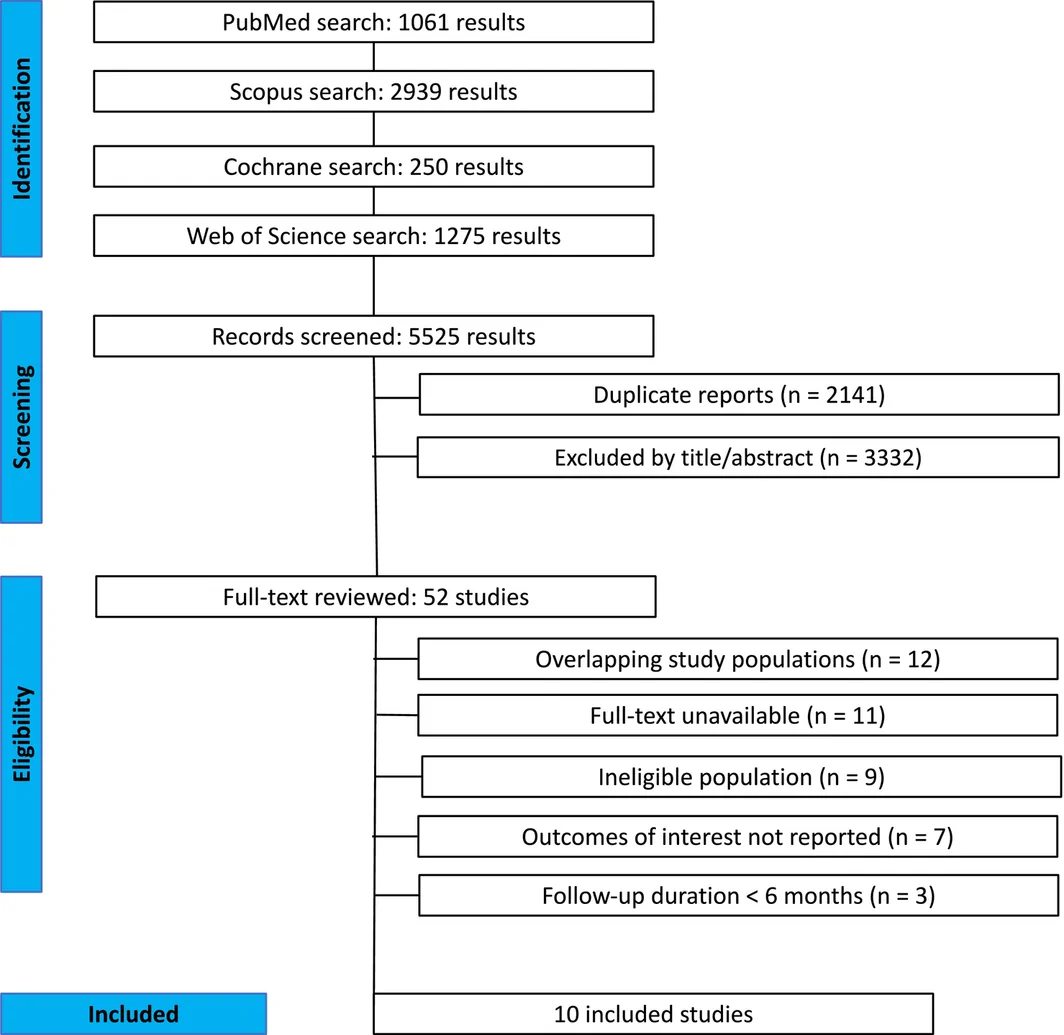
PRISMA flow diagram of study screening and selection.

As illustrated in Figure 1, a total of 5525 records were identified through the database search. After title and abstract screening, 52 articles underwent full‐text assessment for eligibility. A total of 10 studies met the inclusion criteria and were included in the systematic review and meta‐analysis, comprising 41 381 participants [13, 14, 19, 20, 21, 22, 23, 24, 25, 26]. Study characteristics are summarised in Table 1.

**TABLE 1:** Baseline characteristics of the included studies.

Study	Design	Country	Population	Follow‐up (weeks)	Groups	Dose (mg)	Participants	Age (years), mean ± SD	Female, *n* (%)	BMI (kg/m^2^), mean ± SD	Body weight (kg), mean ± SD	T2DM (%)
Frias et al. (SURPASS‐2) [13]	RCT	USA, Argentina, Australia, Brazil, Canada, Israel, Mexico and UK	Adults with T2DM inadequately controlled on metformin, BMI ≥ 25 kg/m^2^	40	**Tir**	5, 10 or 15	470 (5 mg); 469 (10 mg) and 470 (15 mg)	5 mg: 56.3 ± 10.0; 10 mg: 57.2 ± 10.5 and 15 mg: 55.9 ± 10.4	5 mg: 265 (56.4); 10 mg: 231 (49.3) and 15 mg: 256 (54.5)	5 mg: 33.8 ± 6.8; 10 mg: 34.3 ± 6.6 and 15 mg: 34.5 ± 7.1	5 mg: 92.5 ± 21.7; 10 mg: 94.8 ± 22.7 and 15 mg: 93.8 ± 21.8	100.0
					**Sem**	1	469	56.9 ± 10.8	244 (52.0)	34.2 ± 7.1	93.7 ± 21.1	100.0
Heise et al. [19]	RCT	Germany	Adults with T2DM receiving metformin therapy ± another oral antihyperglycaemic agent	28	**Tir**	15	45	61.1 ± 7.1	14 (31.0)	31.28 ± 5.0	94.15 ± 13.9	100.0
					**Sem**	1	44	63.7 ± 5.9	10 (23.0)	30.82 ± 3.8	92.65 ± 14.0	100.0
Rodriguez et al. [20]	Retrospective cohort	USA	Adults with BMI ≥ 27 kg/m^2^ with comorbidity or BMI ≥ 30 kg/m^2^; ±T2DM	48	**Tir**	5	9193	51.9 ± 12.7	6484 (70.5)	39.0 ± 8.1	110 ± 25.7	51.9
					**Sem**	0.5	9192	52.0 ± 13.2	6486 (70.6)	39.1 ± 8.1	110 ± 25.8	52.1
Aronne et al. (SURMOUNT‐5) [14]	RCT	USA and Puerto Rico	Adults with BMI ≥ 27 kg/m^2^ with comorbidity or BMI ≥ 30 kg/m^2^; no diabetes	72	**Tir**	10 or 15 (MTD)	374	45.0 ± 12.9	242 (64.7)	39.4 ± 7.4	112.7 ± 24.8	0
					**Sem**	1.7 or 2.4 (MTD)	376	44.4 ± 12.7	243 (64.6)	39.4 ± 7.7	113.4 ± 26.3	0
Gasoyan et al. [21]	Retrospective cohort	USA	Adults with BMI ≥ 27 kg/m^2^ with comorbidity or BMI ≥ 30 kg/m^2^; no T2DM	48	**Tir**	2.5–15	1772	49.5 ± 12.9	1348 (76.1)	39.4 ± 7.7	111.5 ± 25.3	0
					**Sem**	0.25–2.4	6109	51.8 ± 13.4	4575 (74.9)	39.8 ± 7.8	112.4 ± 25.8	0
Trinh et al. [22]	Retrospective cohort	USA	Adults with overweight/obesity (BMI ≥ 27)	24	**Tir**	NA	109	47.5 ± 11.4	87 (79.8)	44.1 ± 11.3	126.5 ± 33.9	45.0
					**Sem**	NA	836	52.4 ± 12.6	572 (68.4)	40.1 ± 9.0	115.2 ± 28.5	57.4
Ng et al. (SHAPE) [23]	Retrospective cohort	USA	Adults with BMI ≥ 27 kg/m^2^ with comorbidity or BMI ≥ 30 kg/m^2^; no T2DM	48	**Tir**	2.5–15	3122	49.5 ± 10.9	2431 (77.9)	NA	104.9 ± 22.7	0
					**Sem**	0.25–2.4	6794	47.8 ± 10.3	5422 (79.8)	NA	104.5 ± 22.0	0
Bhatti et al. [24]	Retrospective cohort	UAE	Adults with BMI ≥ 27 kg/m^2^ with comorbidity or BMI ≥ 30 kg/m^2^	48	**Tir**	5–15	125	41.8 ± 8.3	89 (71.2)	34.0 ± 5.2	97.5 ± 18.5	4.0
					**Sem**	0.5–2.4	121	40.6 ± 9.7	103 (85.1)	30.5 ± 3.4	86.7 ± 13.9	0
Richards et al. [25]	Retrospective cohort	UK	Adults with overweight/obesity (BMI ≥ 30 kg/m^2^)	48	**Tir**	NA	209	48.2 ± 10.6	173 (82.8)	36.4 ± 6.8	103.5 ± 22.6	0
					**Sem**	NA	130	48.8 ± 10.9	105 (80.8)	37.0 ± 7.9	104.1 ± 25.3	0
le Roux et al. [26]	Retrospective cohort	USA	Adults with BMI ≥ 27 kg/m^2^ with comorbidity or BMI ≥ 30 kg/m^2^	24	**Tir**	10 or 15	1003	48.4 ± 12.2	701 (69.9)	38.3 ± 7.2	104.0 ± 26.0	0
					**Sem**	1.7, 2 or 2.4	1393	49.1 ± 11.9	1020 (73.2)	38.3 ± 7.2	104.0 ± 26.0	0

*Note:* Follow‐up durations originally reported in months were converted approximately to weeks using 4 weeks per month.

Abbreviations: BMI, body mass index; MTD, maximum tolerated dose; NA, not available; RCT, randomised controlled trial; SD, standard deviation; Sem, semaglutide; T2DM, type 2 diabetes mellitus; Tir, tirzepatide; UAE, United Arab Emirates; UK, United Kingdom.

Among the included studies, three were RCTs and seven were retrospective cohort studies. The studies were conducted across multiple geographic regions, including the United States, Europe, the United Kingdom and the United Arab Emirates, with follow‐up durations ranging from 24 to 72 weeks.

Across studies, tirzepatide and semaglutide were administered as once‐weekly subcutaneous injections using study‐specific fixed‐dose, dose‐escalation, maximum tolerated‐dose or variable real‐world dosing regimens. The included studies enrolled a broad population of adults with overweight or obesity, with some cohorts including participants with type 2 diabetes and others specifically enrolling individuals without diabetes. Baseline demographic and clinical characteristics were generally comparable between treatment groups within each study.

### 3.2 Primary Outcome: Percentage Change in Body Weight From Baseline

**FIGURE 2:**
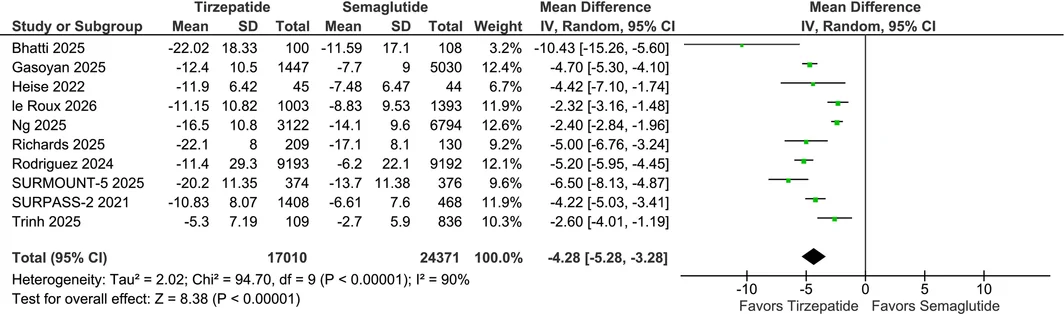
Change in body weight from baseline (%) with tirzepatide compared with semaglutide.

Percentage change in body weight from baseline was reported or derived in all 10 included studies. Across individual studies, mean weight reduction ranged from −22.1% to −5.3% with tirzepatide and from −17.1% to −2.7% with semaglutide. Pooled analysis showed that tirzepatide was associated with a significantly greater reduction in body weight than semaglutide (MD −4.28 percentage points; 95% CI −5.28 to −3.28; *p* < 0.00001), corresponding to an additional mean reduction of approximately 4 percentage points (Figure 2). Substantial heterogeneity was observed (*I* ^2^ = 90%), although all studies favoured tirzepatide.

Prespecified subgroup analyses yielded consistent findings across study design and T2DM status. Tirzepatide produced greater percentage weight reduction in both RCTs (MD −5.03 percentage points; 95% CI −6.62 to −3.43; *I* ^2^ = 67%) and observational cohort studies (MD −4.00 percentage points; 95% CI −5.22 to −2.77; *I* ^2^ = 92%), with no evidence of a difference between study‐design sub‐groups (*p* = 0.32) (Figure S1). The effect also favoured tirzepatide in T2DM‐only populations (MD −4.24 percentage points; 95% CI −5.01 to −3.46; *I* ^2^ = 0%), populations without diabetes (MD −4.05 percentage points; 95% CI −5.47 to −2.63; *I* ^2^ = 93%) and mixed populations with and without T2DM (MD −5.12 percentage points; 95% CI −7.77 to −2.46; *I* ^2^ = 87%), with no evidence of a difference between T2DM‐status subgroups (*p* = 0.78) (Figure S2).

To evaluate the robustness of the primary outcome to risk‐of‐bias concerns in observational evidence, a sensitivity analysis excluding observational studies judged to have serious or critical risk of bias yielded a similar estimate (MD −4.46 percentage points; 95% CI −5.86 to −3.07; *I* ^2^ = 88%) (Figure S3), although heterogeneity remained substantial.

Time‐specific analyses favoured tirzepatide at 24 weeks (MD −2.90 percentage points; 95% CI −3.48 to −2.32; *I* ^2^ = 35%) and at ≥ 48 weeks (MD −5.07 percentage points; 95% CI −6.86 to −3.29; *I* ^2^ = 94%) (Figures S4 and S5). Because some studies contributed data at both time points, no formal comparison between follow‐up periods was performed. Leave‐one‐out sensitivity analyses did not materially alter the pooled estimate for percentage body weight change (Table S1). Visual inspection of the funnel plot showed some asymmetry, mainly driven by one small study with a larger treatment effect favouring tirzepatide. Egger's regression test suggested potential small‐study effects (*p* = 0.022) (Figure S6 and Table S2). These findings should be interpreted cautiously because only 10 studies were included and substantial heterogeneity was present.

### 3.3 Secondary Outcomes

#### 3.3.1 Absolute Change in Body Weight (kg)

**FIGURE 3:**
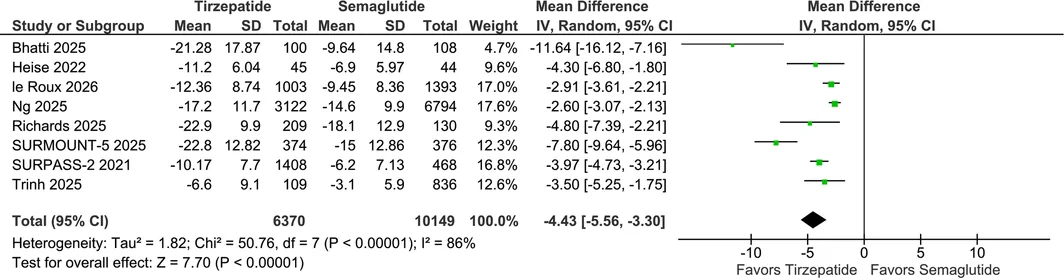
Change in body weight from baseline (kg) with tirzepatide compared with semaglutide.

Absolute change in body weight was reported in eight studies. Mean reductions ranged from −22.9 to −6.6 kg with tirzepatide and from −18.1 to −3.1 kg with semaglutide. In pooled analysis, tirzepatide resulted in a significantly greater absolute weight reduction compared with semaglutide (MD −4.43 kg; 95% CI −5.56 to −3.30; *p* < 0.00001) (Figure 3). Substantial heterogeneity was observed (*I* ^2^ = 86%), although all studies demonstrated a consistent direction of effect favouring tirzepatide.

Subgroup analyses yielded results consistent with the primary analysis. Tirzepatide was associated with greater absolute weight reduction in both RCTs (MD −5.32 kg; 95% CI −7.85 to −2.80; *I* ^2^ = 86%) and observational cohort studies (MD −3.66 kg; 95% CI −4.83 to −2.49; *I* ^2^ = 78%), with no evidence of a difference between study designs (*p* = 0.24) (Figure S7). According to T2DM status, tirzepatide favoured greater weight reduction in T2DM‐only populations (MD −4.00 kg; 95% CI −4.73 to −3.27; *I* ^2^ = 0%) and populations without diabetes (MD −4.23 kg; 95% CI −5.82 to −2.64; *I* ^2^ = 90%). In mixed populations, the estimate also favoured tirzepatide, although the CI crossed the null (MD −7.30 kg; 95% CI −15.26 to 0.66; *I* ^2^ = 91%), with no evidence of differences between T2DM‐status subgroups (*p* = 0.70) (Figure S8). Leave‐one‐out sensitivity analyses did not materially alter the pooled estimate for absolute weight change (Table S3).

#### 3.3.2 Achievement of Weight‐Loss Thresholds

Seven studies reported the proportion of participants achieving clinically relevant weight‐loss thresholds. In pooled analyses, tirzepatide significantly increased the likelihood of achieving higher levels of weight reduction compared with semaglutide. While no statistically significant difference was observed for ≥ 5% weight loss (RR 1.08; 95% CI 0.95–1.23; *p* = 0.24), tirzepatide was associated with progressively greater probabilities of achieving ≥ 10% (RR 1.43; 95% CI 1.20–1.69; *p* < 0.0001), ≥ 15% (RR 1.85; 95% CI 1.45–2.35; *p* < 0.00001) and ≥ 20% weight loss (RR 2.03; 95% CI 1.49–2.75; *p* < 0.00001).

Substantial heterogeneity was observed across analyses (*I* ^2^ range 95%–98%). Forest plots for all categorical weight‐loss thresholds are presented in Figures S9–S12.

#### 3.3.3 Change in Glycated Haemoglobin (HbA1c)

Five studies evaluated change in HbA1c from baseline. Mean HbA1c reductions ranged from −2.31% to −0.50% with tirzepatide and from −1.86% to −0.23% with semaglutide. Pooled analysis demonstrated a significantly greater reduction in HbA1c with tirzepatide (MD −0.29 percentage points; 95% CI −0.44 to −0.14; *p* = 0.0002) (Figure S13). Substantial heterogeneity was observed across studies (*I* ^2^ = 92%), although all studies favoured tirzepatide.

#### 3.3.4 Safety Outcomes

**FIGURE 4:**
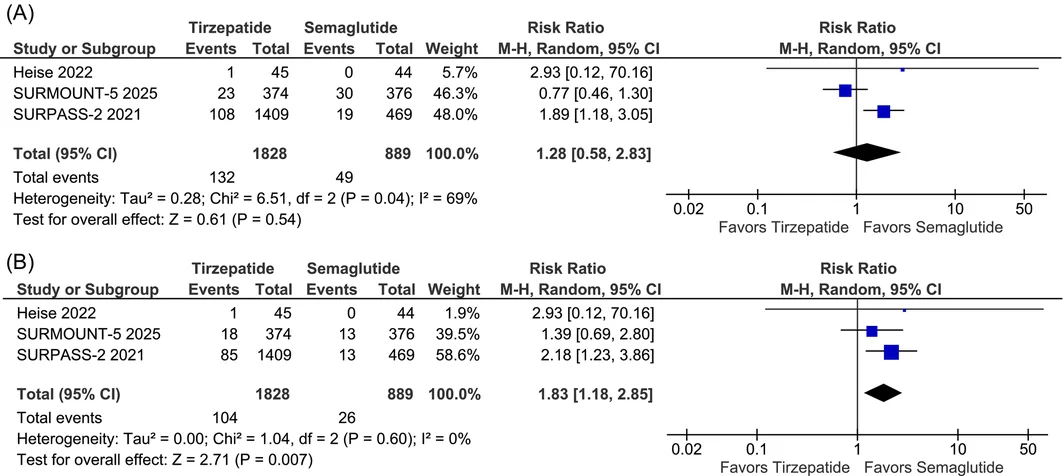
(A) Risk ratio for treatment discontinuation due to adverse events comparing tirzepatide and semaglutide. (B) Risk ratio for serious adverse events comparing tirzepatide and semaglutide.

Three of the included studies reported treatment discontinuation due to adverse events. Discontinuation occurred more frequently among participants treated with tirzepatide (132/1828 [7.2%]) than with semaglutide (49/889 [5.5%]), although the difference was not statistically significant (RR 1.28; 95% CI 0.58–2.83; *p* = 0.54) (Figure 4A). Substantial heterogeneity was observed across studies (*I* ^2^ = 69%).

Serious adverse events were also reported in these three studies and occurred more frequently with tirzepatide (104/1828 [5.7%]) than with semaglutide (26/889 [2.9%]), yielding a significantly higher risk (RR 1.83; 95% CI 1.18–2.85; *p* = 0.007) (Figure 4B). Heterogeneity was not detected (*I* ^2^ = 0%).

For other safety outcomes, including overall adverse events and specific gastrointestinal events (nausea, vomiting, diarrhoea, constipation, abdominal pain, dyspepsia), as well as decreased appetite, pooled RRs did not demonstrate statistically significant differences between treatment groups, with CIs crossing unity (Figures S14–S21).

### 3.4 Quality Assessment

According to the Cochrane Risk of Bias 2 (RoB 2) assessment, the three randomised trials were judged to have low risk of bias across all assessed domains (Figure S22). The randomisation process was considered appropriate, with adequate allocation concealment and balanced baseline characteristics. No major concerns were identified regarding deviations from intended interventions, missing outcome data, outcome measurement or selective reporting.

According to the ROBINS‐I assessment, the observational cohort studies showed an overall risk of bias ranging from moderate to critical (Figure S23), primarily driven by confounding, participant selection, deviations from intended interventions, missing outcome data and selection of the reported result. Two studies were judged to have moderate risk of bias, four serious risk of bias and one critical risk of bias. Although several studies applied statistical adjustment methods, including propensity score‐based approaches, residual and unmeasured confounding could not be excluded.

Given the inclusion of observational studies judged to have serious or critical risk of bias, a sensitivity analysis was performed for the primary outcome excluding these studies. The pooled estimate remained similar and continued to favour tirzepatide, suggesting that the direction of effect was not driven solely by studies at highest risk of bias. Nevertheless, because several observational studies had serious or critical ROBINS‐I ratings, the magnitude of the pooled effect, particularly for observational evidence, should be interpreted cautiously.

## 4 Discussion

This systematic review and meta‐analysis provides the most comprehensive synthesis of the available direct comparative evidence between tirzepatide and semaglutide for the treatment of overweight or obesity. Across 10 studies including 41 381 participants, tirzepatide was consistently associated with greater weight reduction than semaglutide, both in percentage and absolute terms, as well as a higher likelihood of achieving clinically meaningful weight‐loss thresholds. In addition, tirzepatide produced a modest additional reduction in HbA1c but was associated with a higher risk of serious adverse events. Despite substantial heterogeneity across some analyses, the direction of effect was consistent across all studies, supporting the consistency of the findings while not eliminating uncertainty regarding the magnitude of the pooled effect.

The greater weight‐loss efficacy observed with tirzepatide is biologically plausible and likely reflects its dual incretin receptor agonism. While semaglutide selectively activates the GLP‐1 receptor, tirzepatide combines GLP‐1 receptor activation with GIP receptor agonism. Experimental and translational data suggest that this dual mechanism engages complementary neuroendocrine pathways involved in appetite regulation, energy balance and metabolic flexibility, potentially amplifying anorexigenic signalling and contributing to greater reductions in body weight [27].

Our findings are consistent with results from major clinical development programmes. The STEP trials [28, 29, 30, 31, 32] established semaglutide as an effective therapy for weight management, whereas the SURMOUNT programme [33, 34, 35, 36] demonstrated greater weight reductions with tirzepatide, particularly at higher doses. By integrating evidence from both randomised trials and large real‐world cohorts, the present analysis extends these findings and provides a more generalisable estimate of comparative effectiveness in routine clinical practice. Notably, the treatment advantage of tirzepatide was observed at both 24 and ≥ 48 weeks, with a greater magnitude of effect over longer follow‐up.

Previous meta‐analyses have also reported superior weight‐loss efficacy with tirzepatide [15, 37, 38, 39, 40, 41]. However, important methodological differences limit direct comparability. Many prior studies relied predominantly on indirect comparisons across heterogeneous trial populations and study designs. In several cases, these analyses included studies with short follow‐up durations, heterogeneous or poorly defined BMI criteria or highly specific populations such as individuals with type 1 diabetes or prior bariatric surgery, groups typically excluded from randomised trials, which may reduce clinical comparability and limit generalisability of the findings.

In contrast, the present study was restricted to direct head‐to‐head comparisons and applied stricter eligibility criteria, including a minimum follow‐up of 24 weeks and clearly defined populations with overweight or obesity based on BMI thresholds. By prioritising clinically comparable populations and longer follow‐up durations, our analysis provides a more focused and clinically interpretable estimate of comparative effectiveness. However, substantial heterogeneity remained across several outcomes and could only be partially explained by subgroup analyses according to study design, T2DM status and follow‐up duration. Therefore, although the direction of effect consistently favoured tirzepatide, the magnitude of benefit should be interpreted cautiously. Importantly, our findings also extend beyond efficacy by suggesting a higher risk of serious adverse events with tirzepatide in direct comparative analyses, although these safety findings were based on a limited number of studies.

Safety findings warrant careful interpretation. Treatment discontinuation due to adverse events did not differ significantly between tirzepatide and semaglutide, whereas serious adverse events were more frequent with tirzepatide. However, these analyses were based on only three studies, and the analysis of treatment discontinuation showed substantial heterogeneity and a wide confidence interval. Prior network meta‐analyses have generally suggested broadly comparable safety profiles between these agents but relied largely on indirect comparisons across heterogeneous trial networks [40, 42]. By focusing exclusively on direct comparative evidence, the present analysis provides a more clinically interpretable estimate of relative safety. Importantly, overall adverse events and gastrointestinal adverse events, predominantly nausea, vomiting and diarrhoea, did not differ significantly between treatments in pooled analyses. This observation is consistent with findings from the SURPASS and SURMOUNT programmes, in which gastrointestinal symptoms were generally mild‐to‐moderate and dose‐dependent. Variations in dose‐escalation protocols and follow‐up duration across studies may partially explain differences in treatment discontinuation rates.

This study should be interpreted in light of several limitations. Although the number of direct comparative studies included exceeds that of previous syntheses, the overall evidence base remains limited. Large observational cohorts contributed substantially to the total sample size, and residual confounding cannot be excluded despite statistical adjustment and propensity score‐based methods in several studies. Considerable heterogeneity was observed across several efficacy outcomes, likely reflecting differences in baseline BMI, diabetes status, dosing and dose‐escalation regimens, and follow‐up duration. Funnel plot asymmetry and Egger's regression test suggested potential small‐study effects for the primary outcome. In addition, no formal GRADE assessment was performed; therefore, certainty ratings were not assigned to the outcomes. Accordingly, although the direction of effect consistently favoured tirzepatide, confidence in the precise magnitude of the pooled effect is limited, particularly given the substantial heterogeneity, the contribution of observational data and the serious or critical ROBINS‐I ratings observed in several non‐randomised studies.

Dose‐specific analyses were not feasible because dose‐specific outcome data were not consistently reported and several studies used titration schedules, maximum tolerated doses, pooled dose groups or variable real‐world dosing. Categorising studies according to a predominant dose could introduce ecological exposure misclassification; notably, a recent direct‐comparison meta‐analysis using this approach did not demonstrate a clear dose–response pattern and included several comparisons based on few studies or single‐study estimates [15]. The absence of individual participant‐level data also precluded more detailed evaluation of potential effect modifiers. Moreover, the maximum follow‐up duration of 72 weeks limits assessment of long‐term weight‐loss durability and rare safety outcomes, particularly given the known risk of weight regain after discontinuation of incretin‐based therapies [43]. In participants with type 2 diabetes, these therapies may have been prescribed for glycaemic control rather than weight management alone, and the influence of concomitant medications or potential drug interactions could not be systematically evaluated. Finally, real‐world applicability may be influenced by cost, access and insurance coverage, which were not assessed in the included studies.

Despite these limitations, this study has important strengths. By synthesising the largest body of direct comparative evidence currently available and incorporating both randomised and real‐world data, it provides a clinically relevant assessment of the relative effectiveness and safety of tirzepatide and semaglutide. The use of rigorous methodological standards, including PRISMA reporting, prospective protocol registration and structured risk‐of‐bias assessment, further supports the reliability of the findings.

In conclusion, among adults with overweight or obesity, tirzepatide is associated with greater weight reduction and modestly greater glycaemic improvement compared with semaglutide, although these benefits may be accompanied by a higher risk of serious adverse events. These findings highlight the need to balance efficacy and tolerability when selecting incretin‐based therapies. Future studies with longer follow‐up are needed to evaluate the durability of weight loss, long‐term safety and patient‐centred outcomes to better inform individualised treatment decisions.

## Author Contributions

G.P.P. conceived and designed the study, conducted the literature search, independently screened and selected studies, extracted the data, performed the statistical analyses, interpreted the findings and drafted the manuscript. R.F.O. independently screened and selected studies, extracted the data and contributed to drafting and revision of the manuscript. M.M.M. contributed to statistical analyses and drafting and revision of the manuscript. F.P.M.R. contributed to statistical analyses. R.V. critically revised the manuscript for important intellectual content. R.C.P.M. supervised the study, contributed to study design and data interpretation and critically revised the manuscript. All authors reviewed and approved the final manuscript.

## Ethics Statement

This study is a systematic review and meta‐analysis based exclusively on data from previously published studies and publicly available sources. No individual participant data were collected or accessed. Therefore, according to institutional and international guidelines for research ethics, approval from an Institutional Review Board (IRB) or Ethics Committee and informed consent were not required for this study.

## Conflicts of Interest

The authors declare no conflicts of interest.

## Supporting information


**Figure S1:** Forest plot of percentage change in body weight from baseline comparing tirzepatide with semaglutide, stratified by study design.
**Figure S2:** Forest plot of percentage change in body weight from baseline comparing tirzepatide with semaglutide, stratified by study‐level type 2 diabetes status. Mixed T2DM status refers to studies including participants both with and without type 2 diabetes.
**Figure S3:** Sensitivity analysis of percentage change in body weight from baseline comparing tirzepatide with semaglutide, excluding observational studies judged to have serious or critical risk of bias according to ROBINS‐I.
**Figure S4:** Forest plot of percentage change in body weight from baseline comparing tirzepatide with semaglutide at 24 weeks.
**Figure S5:** Forest plot of percentage change in body weight from baseline comparing tirzepatide with semaglutide at ≥ 48 weeks.
**Table S1:** Leave‐one‐out sensitivity analysis for percentage change in body weight from baseline.
**Figure S6:** Funnel plot for percentage change in body weight comparing tirzepatide with semaglutide. The vertical dashed line represents the pooled mean difference.
**Table S2:** Publication bias and small‐study effect assessment for percentage change in body weight.
**Figure S7:** Forest plot of absolute change in body weight comparing tirzepatide with semaglutide, stratified by study design.
**Figure S8:** Forest plot of absolute change in body weight (kg) comparing tirzepatide with semaglutide, stratified by study‐level type 2 diabetes status. Mixed T2DM status refers to studies including participants both with and without type 2 diabetes.
**Table S3:** Leave‐one‐out sensitivity analysis for absolute change in body weight (kg).
**Figure S9:** Proportion of participants achieving ≥ 5% body weight loss from baseline with tirzepatide compared with semaglutide.
**Figure S10:** Proportion of participants achieving ≥ 10% body weight loss from baseline with tirzepatide compared with semaglutide.
**Figure S11:** Proportion of participants achieving ≥ 15% body weight loss from baseline with tirzepatide compared with semaglutide.
**Figure S12:** Proportion of participants achieving ≥ 20% body weight loss from baseline with tirzepatide compared with semaglutide.
**Figure S13:** Mean difference in change in HbA1c (%) from baseline between tirzepatide and semaglutide.
**Figure S14:** Forest plot of overall adverse events comparing tirzepatide with semaglutide. No statistically significant difference was observed between treatment groups (RR 1.00; 95% CI 0.95–1.05; *p* = 0.92). Low heterogeneity was detected (*I* ^2^ = 24%).
**Figure S15:** Forest plot of incidence of nausea comparing tirzepatide with semaglutide. No statistically significant difference was observed (RR 1.01; 95% CI 0.89–1.15; *p* = 0.86; *I* ^2^ = 0%).
**Figure S16:** Forest plot of incidence of vomiting comparing tirzepatide with semaglutide. No statistically significant difference was observed (RR 0.80; 95% CI 0.64–1.01; *p* = 0.06; *I* ^2^ = 0%).
**Figure S17:** Forest plot of incidence of constipation comparing tirzepatide with semaglutide. No statistically significant difference was observed (RR 0.93; 95% CI 0.76–1.14; *p* = 0.48; *I* ^2^ = 0%).
**Figure S18:** Forest plot of incidence of diarrhoea comparing tirzepatide with semaglutide. No statistically significant difference was observed (RR 1.07; 95% CI 0.83–1.36; *p* = 0.61; *I* ^2^ = 33%).
**Figure S19:** Forest plot of incidence of abdominal pain comparing tirzepatide with semaglutide. No statistically significant difference was observed (RR 0.89; 95% CI 0.63–1.24; *p* = 0.48; *I* ^2^ = 0%).
**Figure S20:** Forest plot of incidence of dyspepsia comparing tirzepatide with semaglutide. No statistically significant difference was observed (RR 0.70; 95% CI 0.35–1.41; *p* = 0.32; *I* ^2^ = 74%).
**Figure S21:** Forest plot of incidence of decreased appetite comparing tirzepatide with semaglutide. No statistically significant difference was observed (RR 1.04; 95% CI 0.69–1.58; *p* = 0.84; *I* ^2^ = 62%).
**Figure S22:** Risk of bias assessment according to the Cochrane Risk of Bias 2 (RoB 2) tool.
**Figure S23:** Risk of bias assessment using the ROBINS‐I tool.

## Data Availability

Data sharing not applicable to this article as no datasets were generated or analysed during the current study.
