# Supplementary material for: Comparative Efficacy of Tirzepatide Versus Semaglutide for Weight Loss in Adults With Overweight or Obesity: A Systematic Review and Meta‐Analysis of Head‐to‐Head Studies
Source: Clin Obes. 2026 Aug 30;16(5):e70111. doi: 10.1111/cob.70111 (PMC13527552; doi:10.1111/cob.70111)
Supplement: Supplementary file 1 [file COB-16-e70111-s001.pdf]

Comparative Efficacy of Tirzepatide Versus Semaglutide for Weight Loss in Adults With Overweight or Obesity: A Systematic Review and Meta-Analysis of Head-to-Head Studies

Giovanna Pagani Paccola[1], Roberta Fernandes de Oliveira[1], Matheus Menão Mochetti[2], Fernanda Pataro Marsola Razera[1], Rafael Vecchi[1], Renan Canale Peres Montanher[1]

[1]Faculdade de Medicina de Jaú, Universidade do Oeste Paulista, Jaú, SP, Brazil

[2]Faculdade de Medicina de Bauru, Universidade de São Paulo, Bauru, SP, Brazil

Corresponding author:

Giovanna Pagani Paccola

Email: giovanna.pagani.paccola@gmail.com

Address: Avenida Antônio de Almeida Pacheco, 2945, 2nd Industrial Zone, Jaú, Brazil, ZIP Code 17213-700

Supplementary Material

Supplementary Material 1. Detailed Search Strategy

The systematic search strategy combined controlled vocabulary terms and free-text keywords related to tirzepatide and semaglutide. The following search string was used as the core strategy and adapted for each database: (tirzepatide OR mounjaro OR zepbound OR LY3298176 OR ((GLP-1 OR glucagon-like peptide-1) AND (GIP OR gastric inhibitory polypeptide OR glucose-dependent insulinotropic polypeptide))) AND (semaglutide OR ozempic OR wegovy OR rybelsus OR "glucagon-like peptide-1 receptor agonist" OR "GLP-1 RA" OR "selective GLP-1 receptor agonist" OR "glucagon-like peptide-1 agonist" OR "GLP-1 RAs"). This strategy was adapted for PubMed, Scopus, Web of Science, and the Cochrane Central Register of Controlled Trials using database-specific indexing terms and syntax.

Supplementary Material 2. Detailed Statistical Methods

Continuous outcomes were pooled using mean differences (MDs) with 95% confidence intervals (CIs), whereas dichotomous outcomes were pooled as risk ratios (RRs) with 95% CIs. Random-effects models were used because clinical and methodological heterogeneity was anticipated across studies. Studies reporting time-to-event measures, such as hazard ratios, or incidence rates per person-time were not pooled with studies reporting cumulative event risks, because these effect estimates are not directly comparable. When variability measures were reported as standard errors (SEs) or CIs, these were converted to standard deviations (SDs) according to guidance from the Cochrane Handbook for Systematic Reviews of Interventions.

For studies reporting baseline and follow-up values without SDs for change-from-baseline outcomes, SDs were estimated using reported baseline and follow-up SDs assuming a correlation coefficient, in accordance with Cochrane recommendations. When studies reported absolute weight change but not percentage change, percentage weight reduction was calculated by dividing the mean weight change by the mean baseline body weight and multiplying by 100, with SDs proportionally transformed when appropriate.

When glycated hemoglobin (HbA1c) was reported in mmol/mol, values were converted to percentage units (%) using the internationally accepted IFCC–NGSP conversion formula. For analyses based on change-from-baseline values, the conversion factor was applied to both effect estimates and variability measures prior to pooling.

To avoid unit-of-analysis errors in trials with multiple intervention arms, relevant intervention groups were combined into a single comparison following Cochrane guidance. For the overall pooled analyses, each study contributed only one estimate per outcome. When multiple follow-up time points were reported, data from the longest available

follow-up were used. Adjusted and unadjusted effect estimates were extracted separately when available. Adjusted and unadjusted regression-based estimates were not pooled together. Pooled MDs were calculated from arm-level aggregate outcome estimates, including reported group means, least-squares means, or arm-level estimates from matched analytic populations, with corresponding variability measures for continuous outcomes. Pooled RRs were calculated from event counts and total sample sizes for dichotomous outcomes. When studies reported adjusted estimates from regression models, propensity score–based methods, hazard ratios, or odds ratios, these estimates were extracted separately and used for contextual interpretation rather than combined with aggregate-data estimates.

Prespecified subgroup analyses of percentage and absolute body weight change were performed according to study design, categorized as randomized controlled trials or observational cohort studies, and study-level T2DM status. Studies were classified as T2DM-only populations, populations without diabetes, or mixed populations including participants with and without T2DM. Differences between subgroups were evaluated using tests for subgroup interaction.

A dose-stratified subgroup analysis was not performed because dose-specific outcome data were not consistently available. Several studies used dose-escalation protocols, maximum tolerated doses, pooled multiple-dose arms, or variable real-world dosing, precluding reliable classification into clinically comparable dose categories. Categorizing entire studies according to a predominant dose would represent an ecological study-level comparison and could introduce exposure misclassification.

Separate time-specific analyses of percentage body weight change were conducted at 24 and ≥48 weeks. Because some studies contributed data at both time points, the two

analyses were not independent and no formal test for differences between follow-up periods was performed.

For the primary outcome, a sensitivity analysis excluded observational studies judged to have serious or critical risk of bias according to ROBINS-I. Leave-one-out sensitivity analyses were also performed separately for percentage and absolute body weight change to assess the influence of individual studies on the pooled estimates.

Publication bias and small-study effects were assessed for the primary outcome, because 10 studies contributed to this analysis. Funnel plots were visually inspected for asymmetry, and Egger's regression test was used to evaluate small-study effects. Begg and Mazumdar rank correlation test and trim-and-fill analysis were also performed as exploratory complementary assessments. These analyses were not performed for outcomes including fewer than 10 studies because of limited statistical power and interpretability.

Study or Subgroup	Tirzepatide Mean	Tirzepatide SD	Tirzepatide Total	Semaglutide Mean	Semaglutide SD	Semaglutide Total	Weight	Mean Difference IV, Random, 95% CI
**1.1.1 RCT**								
Heise 2022	-11.9	6.42	45	-7.48	6.47	44	6.7%	-4.42 [-7.10, -1.74]
SURMOUNT-5 2025	-20.2	11.35	374	-13.7	11.38	376	9.6%	-6.50 [-8.13, -4.87]
SURPASS-2 2021	-10.83	8.07	1408	-6.61	7.6	468	11.9%	-4.22 [-5.03, -3.41]
**Subtotal (95% CI)**			**1827**			**888**	**28.3%**	**-5.03 [-6.62, -3.43]**
Heterogeneity: Tau² = 1.29; Chi² = 6.09, df = 2 (P = 0.05); I² = 67%								
Test for overall effect: Z = 6.17 (P < 0.00001)								
**1.1.2 Observational cohort studies**								
Bhatti 2025	-22.02	18.33	100	-11.59	17.1	108	3.2%	-10.43 [-15.26, -5.60]
Gasoyan 2025	-12.4	10.5	1447	-7.7	9	5030	12.4%	-4.70 [-5.30, -4.10]
le Roux 2026	-11.15	10.82	1003	-8.83	9.53	1393	11.9%	-2.32 [-3.16, -1.48]
Ng 2025	-16.5	10.8	3122	-14.1	9.6	6794	12.6%	-2.40 [-2.84, -1.96]
Richards 2025	-22.1	8	209	-17.1	8.1	130	9.2%	-5.00 [-6.76, -3.24]
Rodriguez 2024	-11.4	29.3	9193	-6.2	22.1	9192	12.1%	-5.20 [-5.95, -4.45]
Trinh 2025	-5.3	7.19	109	-2.7	5.9	836	10.3%	-2.60 [-4.01, -1.19]
**Subtotal (95% CI)**			**15183**			**23483**	**71.7%**	**-4.00 [-5.22, -2.77]**
Heterogeneity: Tau² = 2.18; Chi² = 78.89, df = 6 (P < 0.00001); I² = 92%								
Test for overall effect: Z = 6.40 (P < 0.00001)								
**Total (95% CI)**			**17010**			**24371**	**100.0%**	**-4.28 [-5.28, -3.28]**
Heterogeneity: Tau² = 2.02; Chi² = 94.70, df = 9 (P < 0.00001); I² = 90%								
Test for overall effect: Z = 8.38 (P < 0.00001)								
Test for subgroup differences: Chi² = 1.01, df = 1 (P = 0.32), I² = 0.9%								

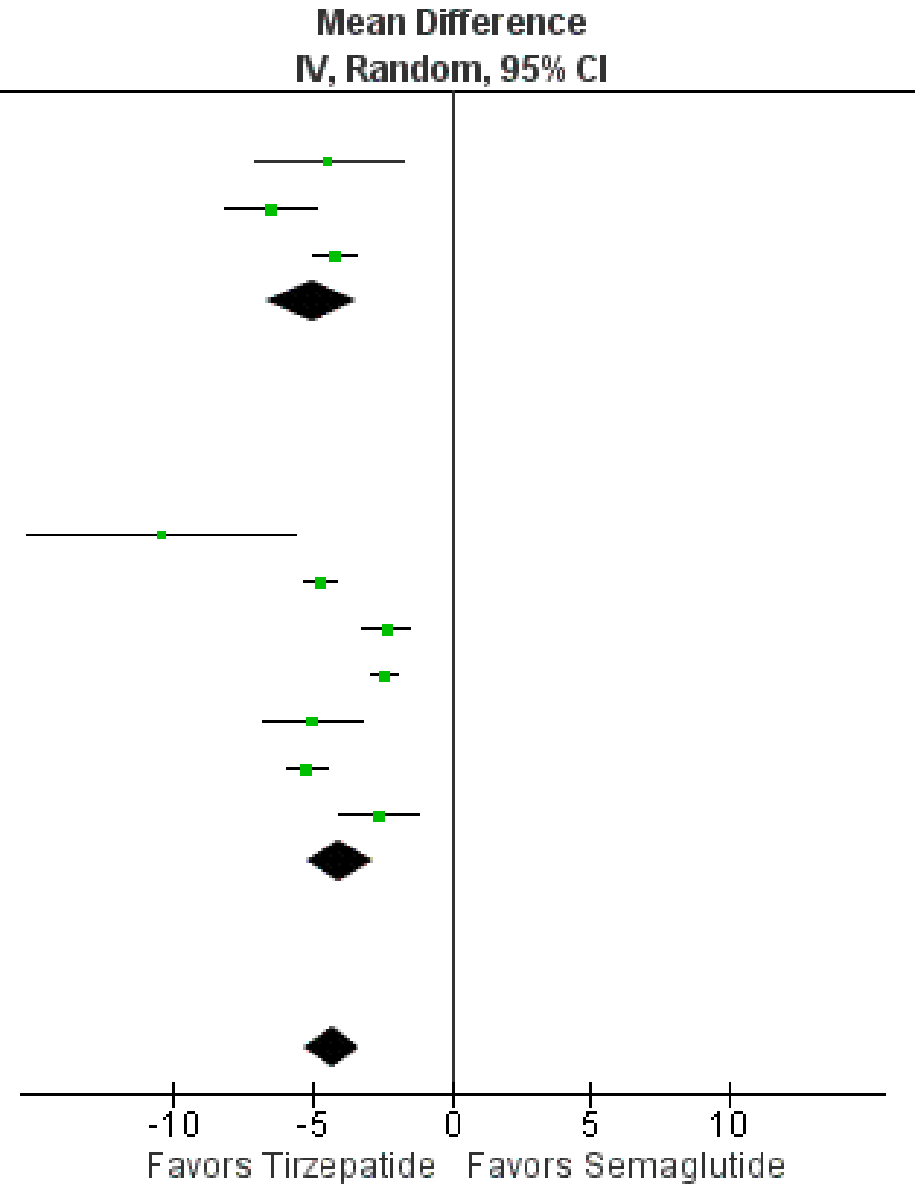

Figure S1: Forest plot of percentage change in body weight from baseline comparing tirzepatide with semaglutide, stratified by study design.

Study or Subgroup	Tirzepatide Mean	Tirzepatide SD	Tirzepatide Total	Semaglutide Mean	Semaglutide SD	Semaglutide Total	Weight	Mean Difference IV, Random, 95% CI
**1.1.1 T2DM-only populations**								
Heise 2022	-11.9	6.42	45	-7.48	6.47	44	6.7%	-4.42 [-7.10, -1.74]
SURPASS-2 2021	-10.83	8.07	1408	-6.61	7.6	468	11.9%	-4.22 [-5.03, -3.41]
**Subtotal (95% CI)**			**1453**			**512**	**18.7%**	**-4.24 [-5.01, -3.46]**
Heterogeneity: Tau² = 0.00; Chi² = 0.02, df = 1 (P = 0.89); I² = 0%								
Test for overall effect: Z = 10.74 (P < 0.00001)								
**1.1.2 Populations without diabetes**								
Gasoyan 2025	-12.4	10.5	1447	-7.7	9	5030	12.4%	-4.70 [-5.30, -4.10]
le Roux 2026	-11.15	10.82	1003	-8.83	9.53	1393	11.9%	-2.32 [-3.16, -1.48]
Ng 2025	-16.5	10.8	3122	-14.1	9.6	6794	12.6%	-2.40 [-2.84, -1.96]
Richards 2025	-22.1	8	209	-17.1	8.1	130	9.2%	-5.00 [-6.76, -3.24]
SURMOUNT-5 2025	-20.2	11.35	374	-13.7	11.38	376	9.6%	-6.50 [-8.13, -4.87]
**Subtotal (95% CI)**			**6155**			**13723**	**55.7%**	**-4.05 [-5.47, -2.63]**
Heterogeneity: Tau² = 2.29; Chi² = 60.82, df = 4 (P < 0.00001); I² = 93%								
Test for overall effect: Z = 5.59 (P < 0.00001)								
**1.1.3 Mixed T2DM status**								
Bhatti 2025	-22.02	18.33	100	-11.59	17.1	108	3.2%	-10.43 [-15.26, -5.60]
Rodriguez 2024	-11.4	29.3	9193	-6.2	22.1	9192	12.1%	-5.20 [-5.95, -4.45]
Trinh 2025	-5.3	7.19	109	-2.7	5.9	836	10.3%	-2.60 [-4.01, -1.19]
**Subtotal (95% CI)**			**9402**			**10136**	**25.6%**	**-5.12 [-7.77, -2.46]**
Heterogeneity: Tau² = 4.15; Chi² = 15.66, df = 2 (P = 0.0004); I² = 87%								
Test for overall effect: Z = 3.78 (P = 0.0002)								
**Total (95% CI)**			**17010**			**24371**	**100.0%**	**-4.28 [-5.28, -3.28]**
Heterogeneity: Tau² = 2.02; Chi² = 94.70, df = 9 (P < 0.00001); I² = 90%								
Test for overall effect: Z = 8.38 (P < 0.00001)								
Test for subgroup differences: Chi² = 0.49, df = 2 (P = 0.78), I² = 0%								

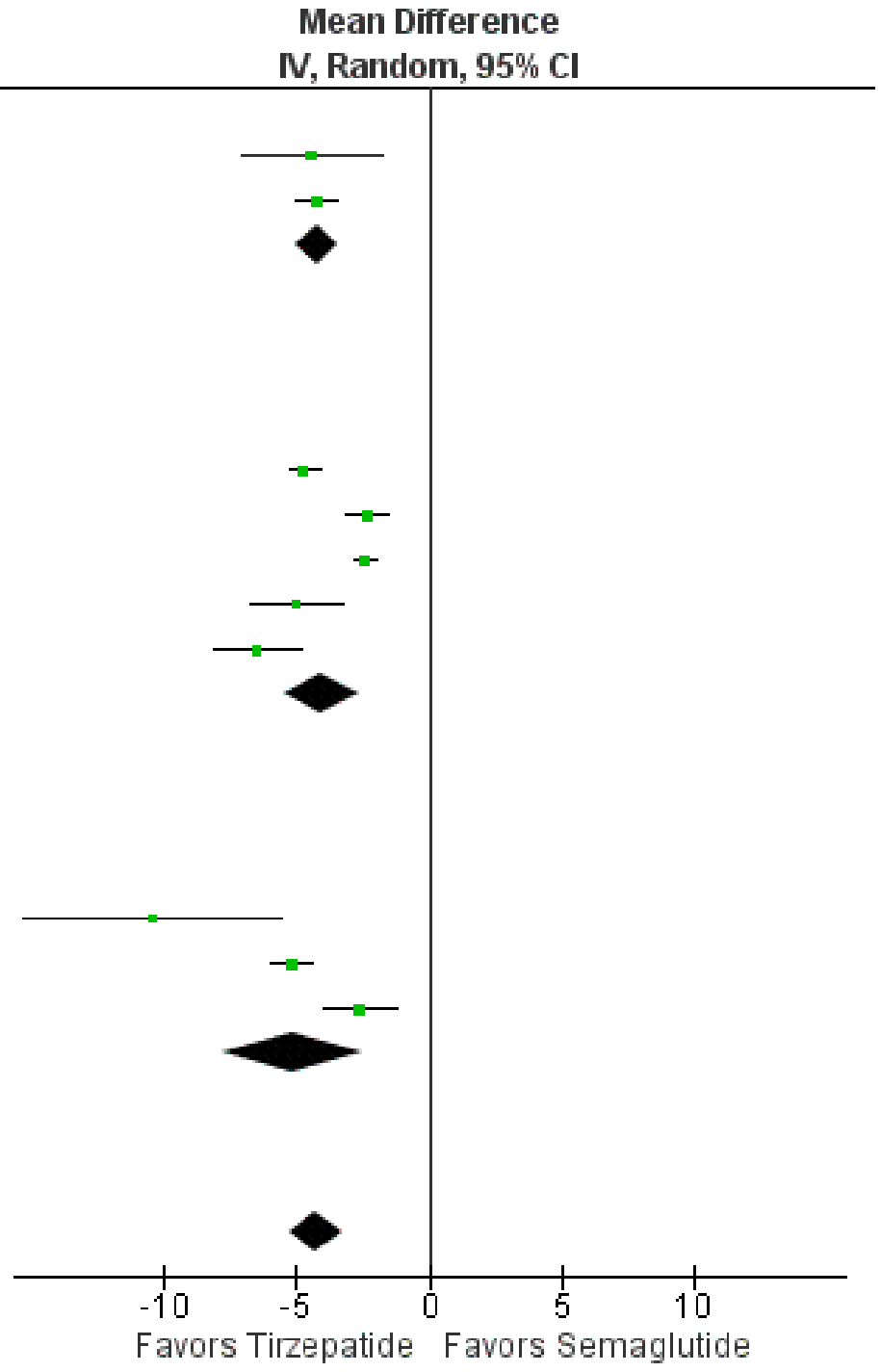

Figure S2: Forest plot of percentage change in body weight from baseline comparing tirzepatide with semaglutide, stratified by study-level type 2 diabetes status. Mixed T2DM status refers to studies including participants both with and without type 2 diabetes.

Study or Subgroup	Tirzepatide Mean	Tirzepatide SD	Tirzepatide Total	Semaglutide Mean	Semaglutide SD	Semaglutide Total	Weight	Mean Difference IV, Random, 95% CI
Heise 2022	-11.9	6.42	45	-7.48	6.47	44	12.9%	-4.42 [-7.10, -1.74]
le Roux 2026	-11.15	10.82	1003	-8.83	9.53	1393	22.7%	-2.32 [-3.16, -1.48]
Rodriguez 2024	-11.4	29.3	9193	-6.2	22.1	9192	23.1%	-5.20 [-5.95, -4.45]
SURMOUNT-5 2025	-20.2	11.35	374	-13.7	11.38	376	18.5%	-6.50 [-8.13, -4.87]
SURPASS-2 2021	-10.83	8.07	1408	-6.61	7.6	468	22.8%	-4.22 [-5.03, -3.41]
**Total (95% CI)**			**12023**			**11473**	**100.0%**	**-4.46 [-5.86, -3.07]**

Heterogeneity: Tau² = 2.06; Chi² = 33.96, df = 4 (P < 0.00001); I² = 88%
Test for overall effect: Z = 6.26 (P < 0.00001)

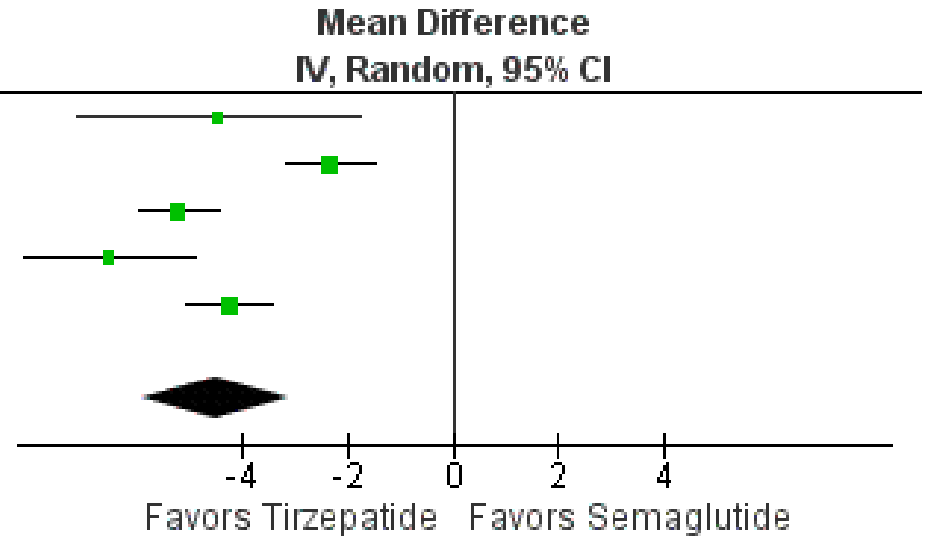

Figure S3: Sensitivity analysis of percentage change in body weight from baseline comparing tirzepatide with semaglutide, excluding observational studies judged to have serious or critical risk of bias according to ROBINS-I.

	Tirzepatide			Semaglutide				Mean Difference
Study or Subgroup	Mean	SD	Total	Mean	SD	Total	Weight	IV, Random, 95% CI
Bhatti 2025	-10.17	17.82	100	-9.09	13.99	108	1.7%	-1.08 [-5.46, 3.30]
Heise 2022	-11.9	6.42	45	-7.48	6.47	44	4.4%	-4.42 [-7.10, -1.74]
le Roux 2026	-11.15	10.82	1003	-8.83	9.53	1393	26.7%	-2.32 [-3.16, -1.48]
Rodriguez 2024	-8.2	9.8	9193	-5	7.3	9192	54.0%	-3.20 [-3.45, -2.95]
Trinh 2025	-5.3	7.19	109	-2.7	5.9	836	13.2%	-2.60 [-4.01, -1.19]
**Total (95% CI)**			**10450**			**11573**	**100.0%**	**-2.90 [-3.48, -2.32]**

Heterogeneity: $Tau^2 = 0.15$; $Chi^2 = 6.17$, df = 4 (P = 0.19); $I^2 = 35\%$

Test for overall effect: Z = 9.81 (P < 0.00001)

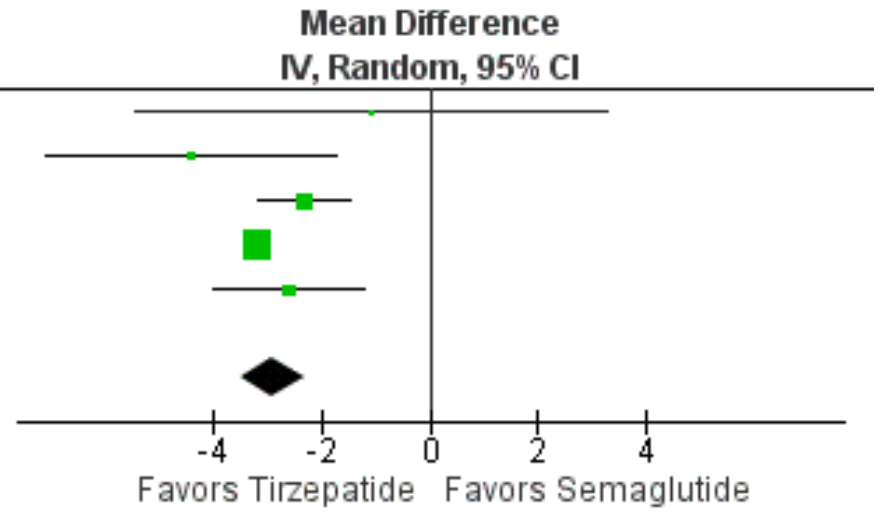

Figure S4: Forest plot of percentage change in body weight from baseline comparing tirzepatide with semaglutide at 24 weeks.

Study or Subgroup	Tirzepatide Mean	Tirzepatide SD	Tirzepatide Total	Semaglutide Mean	Semaglutide SD	Semaglutide Total	Weight	Mean Difference IV, Random, 95% CI
Bhatti 2025	-22.02	18.33	100	-11.59	17.1	108	8.9%	-10.43 [-15.26, -5.60]
Gasoyan 2025	-12.4	10.5	1447	-7.7	9	5030	24.7%	-4.70 [-5.30, -4.10]
Ng 2025	-16.5	10.8	3122	-14.1	9.6	6794	25.0%	-2.40 [-2.84, -1.96]
Richards 2025	-22.1	8	209	-17.1	8.1	130	20.4%	-5.00 [-6.76, -3.24]
SURMOUNT-5 2025	-20.2	11.35	374	-13.7	11.38	376	21.0%	-6.50 [-8.13, -4.87]
**Total (95% CI)**			**5252**			**12438**	**100.0%**	**-5.07 [-6.86, -3.29]**

Heterogeneity: Tau² = 3.26; Chi² = 63.07, df = 4 (P < 0.00001); I² = 94%
Test for overall effect: Z = 5.57 (P < 0.00001)

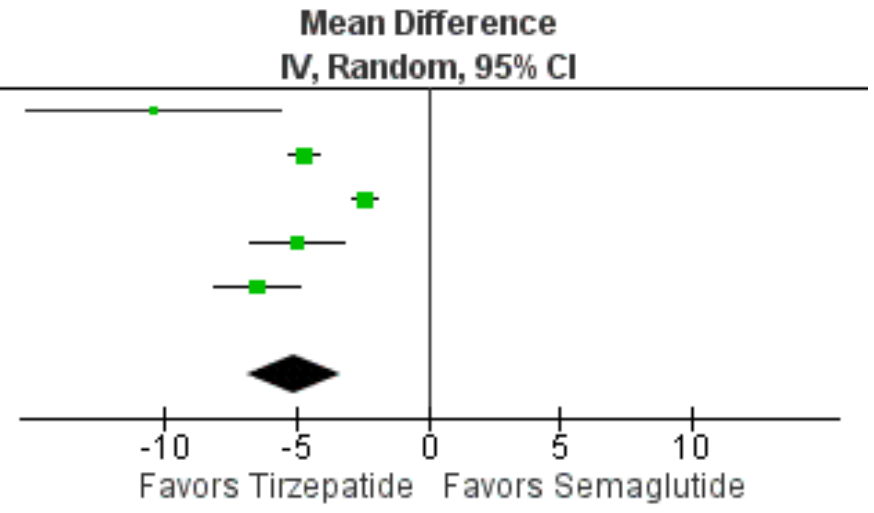

Figure S5: Forest plot of percentage change in body weight from baseline comparing tirzepatide with semaglutide at ≥48 weeks.

Table S1: Leave-one-out sensitivity analysis for percentage change in body weight from baseline.

Study removed	Pooled MD	95% CI	I^2
None, overall analysis	-4.28	-5.28 to -3.28	90%
Bhatti 2025	-4.07	-5.06 to -3.09	91%
Gasoyan 2025	-4.25	-5.37 to -3.13	90%
Heise 2022	-4.28	-5.32 to -3.23	92%
le Roux 2026	-4.55	-5.64 to -3.47	91%
Ng 2025	-4.50	-5.45 to -3.56	83%
Richards 2025	-4.21	-5.27 to -3.15	91%
Rodriguez 2024	-4.15	-5.19 to -3.10	89%
SURMOUNT-5 2025	-4.03	-5.04 to -3.02	90%
SURPASS-2 2021	-4.32	-5.46 to -3.18	91%
Trinh 2025	-4.48	-5.56 to -3.41	91%

MD: mean difference; CI: confidence interval. Negative values favor tirzepatide.

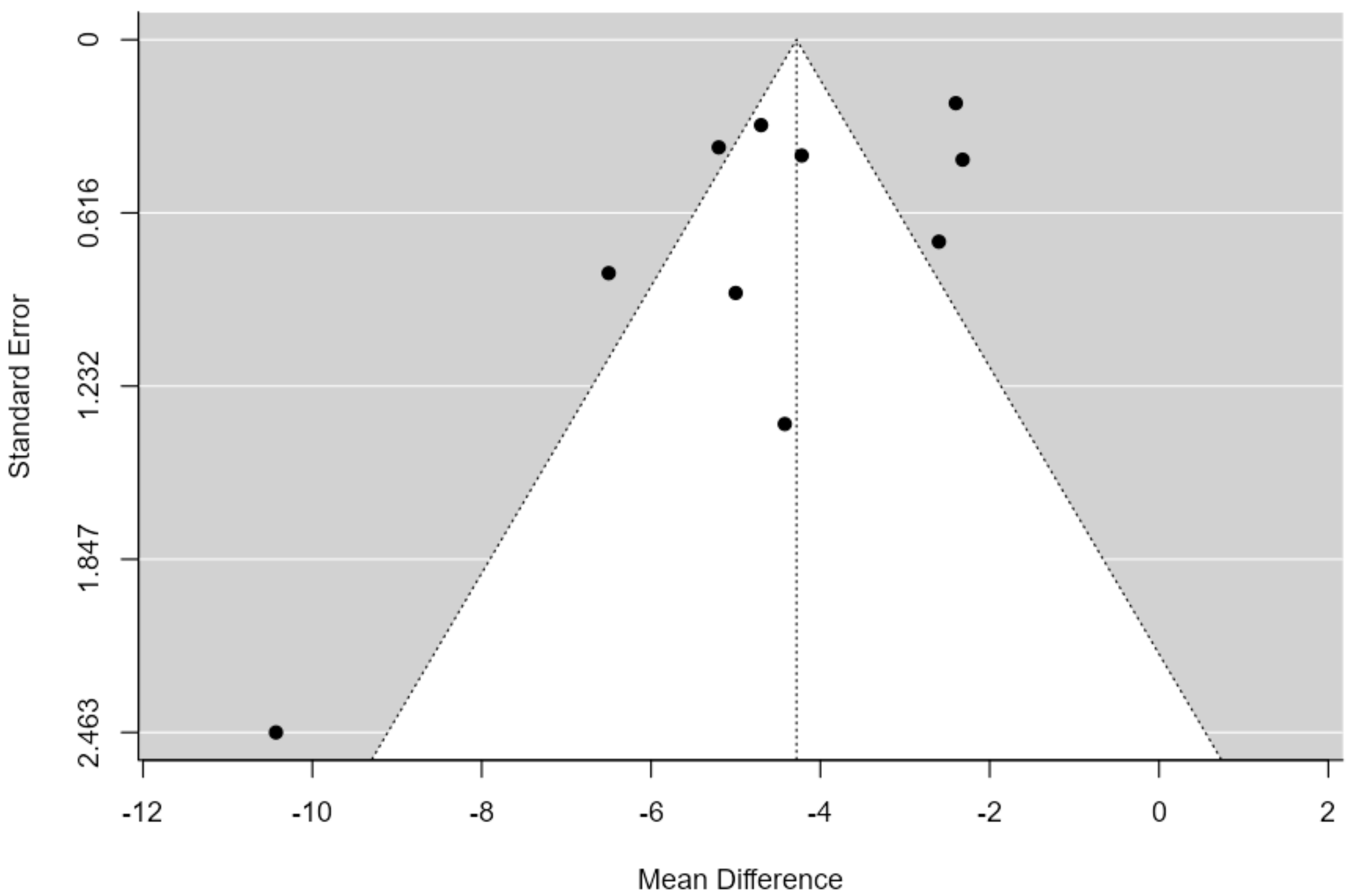

Figure S6: Funnel plot for percentage change in body weight comparing tirzepatide with semaglutide. The vertical dashed line represents the pooled mean difference.

Table S2: Publication bias and small-study effect assessment for percentage change in body weight.

**Test**	**Value**	**p-value**
Begg and Mazumdar rank correlation	-0.067	0.862
Egger’s regression	-2.288	0.022
Trim-and-fill number of studies	1	NA

NA: not applicable. Publication bias analyses were performed only for the primary outcome because 10 studies contributed to this analysis.

Study or Subgroup	Tirzepatide Mean	Tirzepatide SD	Tirzepatide Total	Semaglutide Mean	Semaglutide SD	Semaglutide Total	Weight	Mean Difference IV, Random, 95% CI
**3.1.1 RCT**								
Heise 2022	-11.2	6.04	45	-6.9	5.97	44	9.6%	-4.30 [-6.80, -1.80]
SURMOUNT-5 2025	-22.8	12.82	374	-15	12.86	376	12.3%	-7.80 [-9.64, -5.96]
SURPASS-2 2021	-10.17	7.7	1408	-6.2	7.13	468	16.8%	-3.97 [-4.73, -3.21]
**Subtotal (95% CI)**			**1827**			**888**	**38.7%**	**-5.32 [-7.85, -2.80]**
Heterogeneity: Tau² = 4.17; Chi² = 14.27, df = 2 (P = 0.0008); I² = 86%								
Test for overall effect: Z = 4.13 (P < 0.0001)								
**3.1.2 Observational cohort studies**								
Bhatti 2025	-21.28	17.87	100	-9.64	14.8	108	4.7%	-11.64 [-16.12, -7.16]
le Roux 2026	-12.36	8.74	1003	-9.45	8.36	1393	17.0%	-2.91 [-3.61, -2.21]
Ng 2025	-17.2	11.7	3122	-14.6	9.9	6794	17.6%	-2.60 [-3.07, -2.13]
Richards 2025	-22.9	9.9	209	-18.1	12.9	130	9.3%	-4.80 [-7.39, -2.21]
Trinh 2025	-6.6	9.1	109	-3.1	5.9	836	12.6%	-3.50 [-5.25, -1.75]
**Subtotal (95% CI)**			**4543**			**9261**	**61.3%**	**-3.66 [-4.83, -2.49]**
Heterogeneity: Tau² = 1.06; Chi² = 18.60, df = 4 (P = 0.0009); I² = 78%								
Test for overall effect: Z = 6.13 (P < 0.00001)								
**Total (95% CI)**			**6370**			**10149**	**100.0%**	**-4.43 [-5.56, -3.30]**
Heterogeneity: Tau² = 1.82; Chi² = 50.76, df = 7 (P < 0.00001); I² = 86%								
Test for overall effect: Z = 7.70 (P < 0.00001)								
Test for subgroup differences: Chi² = 1.37, df = 1 (P = 0.24), I² = 27.0%								

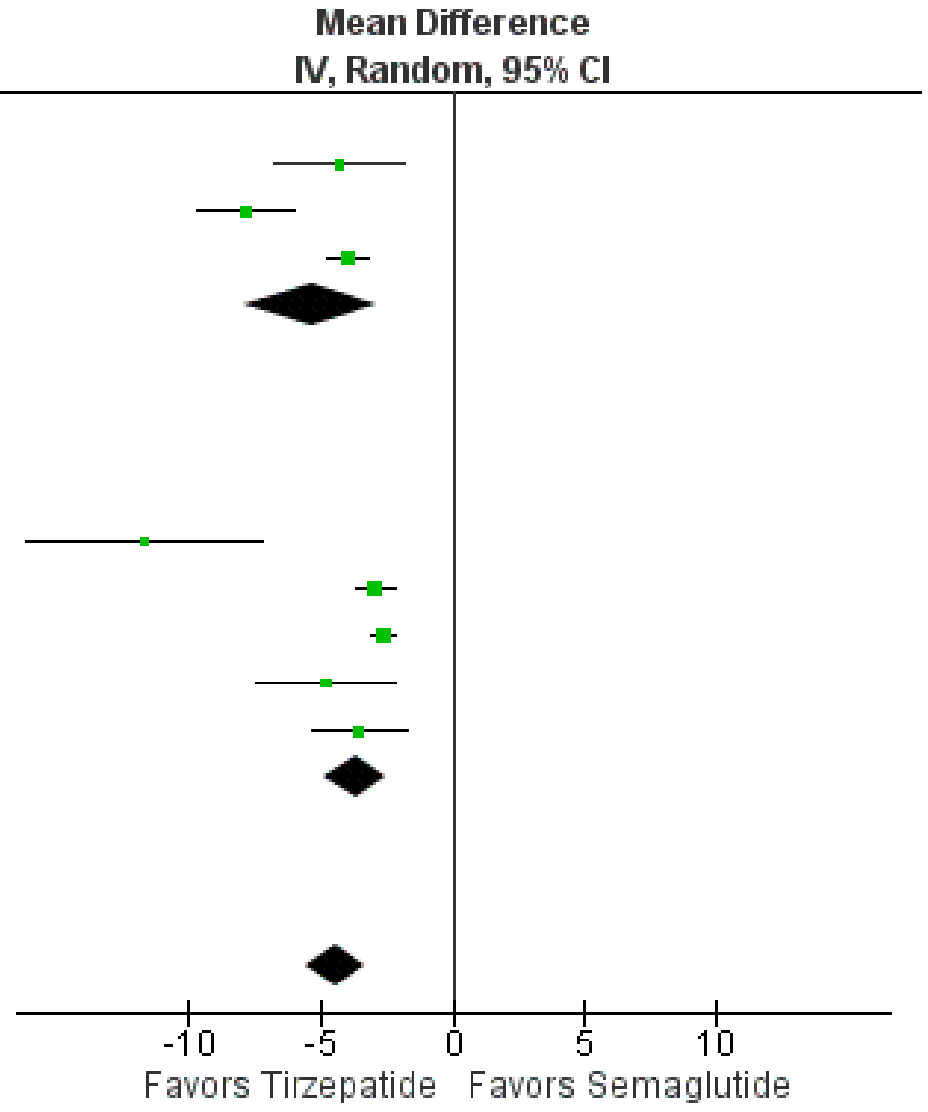

Figure S7: Forest plot of absolute change in body weight comparing tirzepatide with semaglutide, stratified by study design.

	Tirzepatide			Semaglutide				Mean Difference
**Study or Subgroup**	**Mean**	**SD**	**Total**	**Mean**	**SD**	**Total**	**Weight**	**IV, Random, 95% CI**
**3.1.1 T2DM-only populations**								
SURPASS-2 2021	-10.17	7.7	1408	-6.2	7.13	468	16.8%	-3.97 [-4.73, -3.21]
Heise 2022	-11.2	6.04	45	-6.9	5.97	44	9.6%	-4.30 [-6.80, -1.80]
**Subtotal (95% CI)**			**1453**			**512**	**26.4%**	**-4.00 [-4.73, -3.27]**
Heterogeneity: Tau² = 0.00; Chi² = 0.06, df = 1 (P = 0.80); I² = 0%								
Test for overall effect: Z = 10.77 (P < 0.00001)								
**3.1.2 Populations without diabetes**								
SURMOUNT-5 2025	-22.8	12.82	374	-15	12.86	376	12.3%	-7.80 [-9.64, -5.96]
Richards 2025	-22.9	9.9	209	-18.1	12.9	130	9.3%	-4.80 [-7.39, -2.21]
Ng 2025	-17.2	11.7	3122	-14.6	9.9	6794	17.6%	-2.60 [-3.07, -2.13]
le Roux 2026	-12.36	8.74	1003	-9.45	8.36	1393	17.0%	-2.91 [-3.61, -2.21]
**Subtotal (95% CI)**			**4708**			**8693**	**56.2%**	**-4.23 [-5.82, -2.64]**
Heterogeneity: Tau² = 2.08; Chi² = 30.82, df = 3 (P < 0.00001); I² = 90%								
Test for overall effect: Z = 5.21 (P < 0.00001)								
**3.1.3 Mixed T2DM status**								
Trinh 2025	-6.6	9.1	109	-3.1	5.9	836	12.6%	-3.50 [-5.25, -1.75]
Bhatti 2025	-21.28	17.87	100	-9.64	14.8	108	4.7%	-11.64 [-16.12, -7.16]
**Subtotal (95% CI)**			**209**			**944**	**17.3%**	**-7.30 [-15.26, 0.66]**
Heterogeneity: Tau² = 30.12; Chi² = 11.00, df = 1 (P = 0.0009); I² = 91%								
Test for overall effect: Z = 1.80 (P = 0.07)								
**Total (95% CI)**			**6370**			**10149**	**100.0%**	**-4.43 [-5.56, -3.30]**
Heterogeneity: Tau² = 1.82; Chi² = 50.76, df = 7 (P < 0.00001); I² = 86%								
Test for overall effect: Z = 7.70 (P < 0.00001)								
Test for subgroup differences: Chi² = 0.71, df = 2 (P = 0.70), I² = 0%								

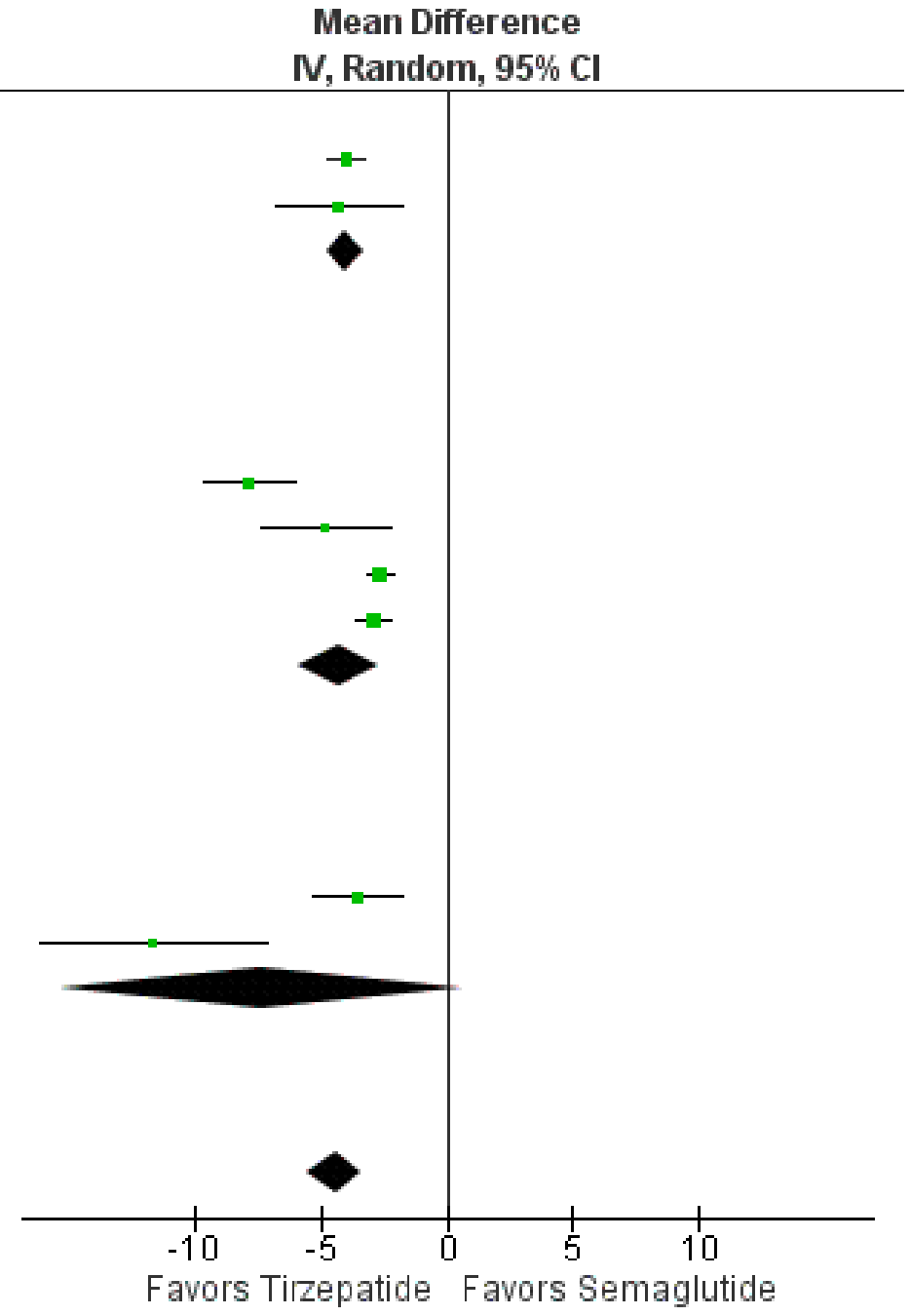

Figure S8: Forest plot of absolute change in body weight (kg) comparing tirzepatide with semaglutide, stratified by study-level type 2 diabetes status. Mixed T2DM status refers to studies including participants both with and without type 2 diabetes.

Table S3: Leave-one-out sensitivity analysis for absolute change in body weight (kg).

Study removed	Pooled MD	95% CI	I^2
None, overall analysis	-4.43	-5.56 to -3.30	86%
Bhatti 2025	-4.02	-5.03 to -3.00	84%
Heise 2022	-4.46	-5.66 to -3.25	88%
le Roux 2026	-4.89	-6.36 to -3.41	88%
Ng 2025	-4.92	-6.33 to -3.51	84%
Richards 2025	-4.40	-5.59 to -3.20	88%
SURMOUNT-5 2025	-3.74	-4.66 to -2.81	77%
SURPASS-2 2021	-4.63	-6.02 to -3.25	87%
Trinh 2025	-4.60	-5.85 to -3.35	88%

MD: mean difference; CI: confidence interval. Negative values favor tirzepatide.

Study or Subgroup	Tirzepatide Events	Tirzepatide Total	Semaglutide Events	Semaglutide Total	Weight	Risk Ratio M-H, Random, 95% CI
Gasoyan 2025	1057	1447	2877	5030	21.5%	1.28 [1.23, 1.33]
le Roux 2026	860	1003	1048	1393	21.5%	1.14 [1.10, 1.19]
Ng 2025	2691	3122	5714	6794	21.8%	1.02 [1.01, 1.04]
SURPASS-2 2021	1094	1408	270	468	20.1%	1.35 [1.24, 1.46]
Trinh 2025	56	109	675	836	15.1%	0.64 [0.53, 0.77]
**Total (95% CI)**		**7089**		**14521**	**100.0%**	**1.08 [0.95, 1.23]**
Total events	5758		10584			

Heterogeneity: $Tau^2 = 0.02$; $Chi^2 = 180.50$, df = 4 (P < 0.00001); $I^2 = 98\%$
Test for overall effect: Z = 1.17 (P = 0.24)

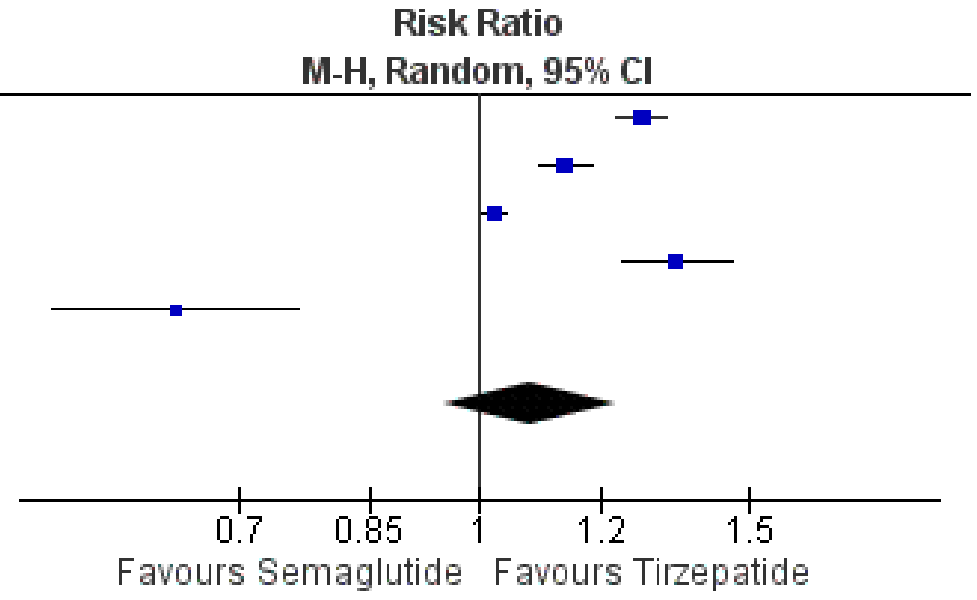

Figure S9: Proportion of participants achieving ≥5% body weight loss from baseline with tirzepatide compared with semaglutide.

Study or Subgroup	Tirzepatide Events	Tirzepatide Total	Semaglutide Events	Semaglutide Total	Weight	Risk Ratio M-H, Random, 95% CI
Gasoyan 2025	813	1447	1801	5030	15.5%	1.57 [1.48, 1.66]
le Roux 2026	592	1003	529	1393	15.2%	1.55 [1.43, 1.69]
Ng 2025	2304	3122	4536	6794	15.7%	1.11 [1.08, 1.14]
Richards 2025	199	209	108	130	15.2%	1.15 [1.05, 1.25]
Rodriguez 2024	0	0	0	0		Not estimable
SURMOUNT-5 2025	304	374	227	376	15.1%	1.35 [1.22, 1.48]
SURPASS-2 2021	709	1408	117	468	13.8%	2.01 [1.71, 2.38]
Trinh 2025	28	109	139	836	9.4%	1.54 [1.08, 2.20]
**Total (95% CI)**		**7672**		**15027**	**100.0%**	**1.43 [1.20, 1.69]**
Total events	4949		7457			

Heterogeneity: Tau² = 0.05; Chi² = 218.07, df = 6 (P < 0.00001); I² = 97%

Test for overall effect: Z = 4.07 (P < 0.0001)

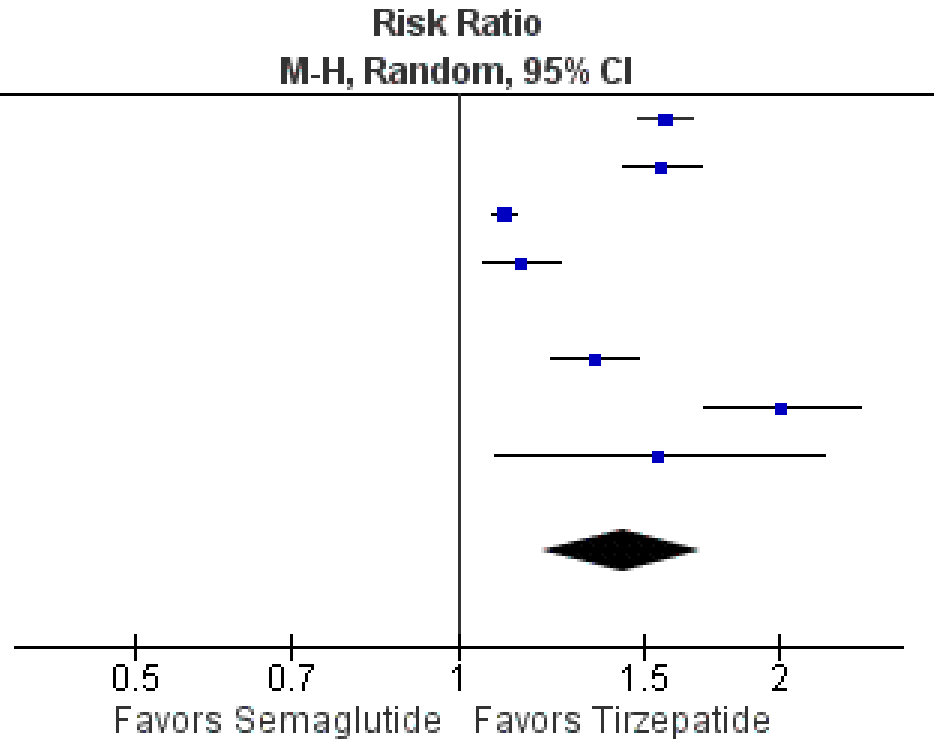

Figure S10: Proportion of participants achieving ≥10% body weight loss from baseline with tirzepatide compared with semaglutide.

Study or Subgroup	Tirzepatide Events	Tirzepatide Total	Semaglutide Events	Semaglutide Total	Weight	Risk Ratio M-H, Random, 95% CI
Gasoyan 2025	574	1447	1012	5030	16.0%	1.97 [1.81, 2.14]
le Roux 2026	313	1003	195	1393	15.3%	2.23 [1.90, 2.61]
Ng 2025	1749	3122	3045	6794	16.3%	1.25 [1.20, 1.30]
Richards 2025	175	209	73	130	15.2%	1.49 [1.27, 1.76]
SURMOUNT-5 2025	241	374	151	376	15.5%	1.60 [1.39, 1.85]
SURPASS-2 2021	382	1408	40	468	12.9%	3.17 [2.33, 4.32]
Trinh 2025	14	109	52	836	8.8%	2.06 [1.18, 3.60]
**Total (95% CI)**		**7672**		**15027**	**100.0%**	**1.85 [1.45, 2.35]**
Total events	3448		4568			

Heterogeneity: Tau² = 0.09; Chi² = 164.19, df = 6 (P < 0.00001); I² = 96%
Test for overall effect: Z = 4.98 (P < 0.00001)

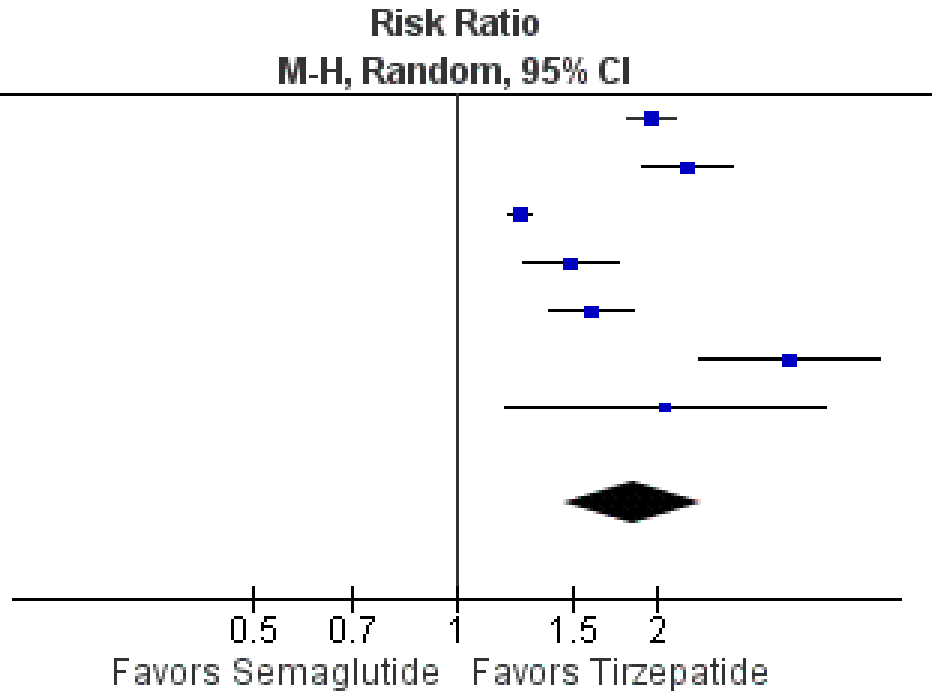

Figure S11: Proportion of participants achieving ≥15% body weight loss from baseline with tirzepatide compared with semaglutide.

Study or Subgroup	Tirzepatide Events	Tirzepatide Total	Semaglutide Events	Semaglutide Total	Weight	Risk Ratio M-H, Random, 95% CI
Gasoyan 2025	344	1447	508	5030	26.4%	2.35 [2.08, 2.66]
le Roux 2026	113	1003	53	1393	21.4%	2.96 [2.16, 4.06]
Ng 2025	1169	3122	1721	6794	27.3%	1.48 [1.39, 1.57]
SURMOUNT-5 2025	181	374	103	376	24.9%	1.77 [1.45, 2.15]
**Total (95% CI)**		**5946**		**13593**	**100.0%**	**2.03 [1.49, 2.75]**
Total events	1807		2385			

Heterogeneity: Tau² = 0.09; Chi² = 58.06, df = 3 (P < 0.00001); I² = 95%
Test for overall effect: Z = 4.51 (P < 0.00001)

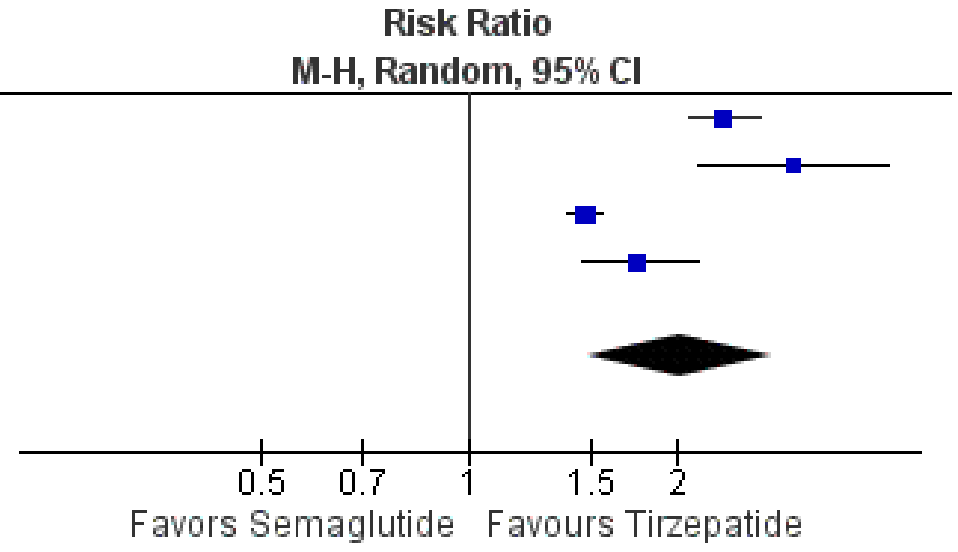

Figure S12: Proportion of participants achieving ≥20% body weight loss from baseline with tirzepatide compared with semaglutide.

Study or Subgroup	Tirzepatide Mean	Tirzepatide SD	Tirzepatide Total	Semaglutide Mean	Semaglutide SD	Semaglutide Total	Weight	Mean Difference IV, Random, 95% CI
Bhatti 2025	-0.6	0.43	100	-0.23	0.37	108	20.5%	-0.37 [-0.48, -0.26]
Heise 2022	-2.05	0.55	45	-1.64	0.54	44	15.2%	-0.41 [-0.64, -0.18]
le Roux 2026	-0.52	0.31	69	-0.37	0.31	106	21.1%	-0.15 [-0.24, -0.06]
SURMOUNT-5 2025	-0.5	0.3	374	-0.39	0.3	376	22.6%	-0.11 [-0.15, -0.07]
SURPASS-2 2021	-2.31	1.04	1408	-1.86	1.04	468	20.6%	-0.45 [-0.56, -0.34]
**Total (95% CI)**			**1996**			**1102**	**100.0%**	**-0.29 [-0.44, -0.14]**

Heterogeneity: $Tau^2 = 0.03$; $Chi^2 = 49.80$, df = 4 ($P < 0.00001$); $I^2 = 92\%$

Test for overall effect: $Z = 3.73$ ($P = 0.0002$)

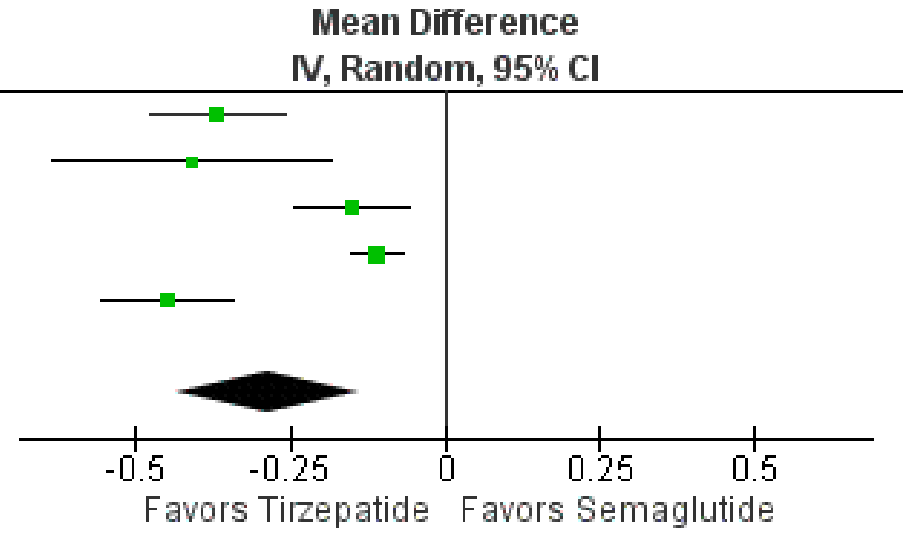

Figure S13: Mean difference in change in HbA1c (%) from baseline between tirzepatide and semaglutide.

Study or Subgroup	Tirzepatide Events	Tirzepatide Total	Semaglutide Events	Semaglutide Total	Weight	Risk Ratio M-H, Random, 95% CI
Heise 2022	43	45	43	44	33.0%	0.98 [0.90, 1.06]
SURMOUNT-5 2025	287	374	297	376	33.7%	0.97 [0.90, 1.05]
SURPASS-2 2021	945	1409	301	469	33.3%	1.05 [0.97, 1.13]
**Total (95% CI)**		**1828**		**889**	**100.0%**	**1.00 [0.95, 1.05]**
Total events	1275		641			

Heterogeneity: Tau² = 0.00; Chi² = 2.63, df = 2 (P = 0.27); I² = 24%

Test for overall effect: Z = 0.09 (P = 0.92)

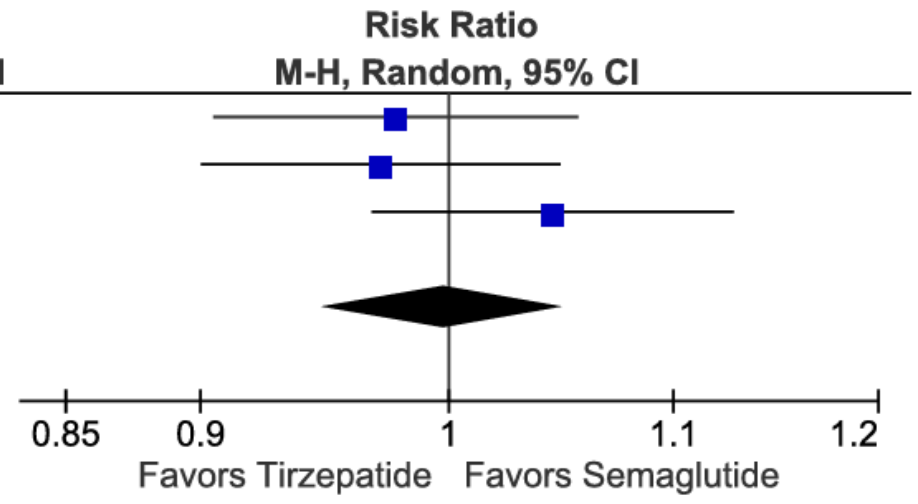

Figure S14: Forest plot of overall adverse events comparing tirzepatide with semaglutide. No statistically significant difference was observed between treatment groups (RR 1.00; 95% CI 0.95–1.05; p = 0.92). Low heterogeneity was detected (I² = 24%).

	Tirzepatide		Semaglutide			Risk Ratio
**Study or Subgroup**	**Events**	**Total**	**Events**	**Total**	**Weight**	**M-H, Random, 95% CI**
Heise 2022	11	45	13	44	3.5%	0.83 [0.42, 1.64]
SURMOUNT-5 2025	163	374	167	376	62.9%	0.98 [0.83, 1.15]
SURPASS-2 2021	276	1409	84	469	33.7%	1.09 [0.88, 1.36]
**Total (95% CI)**		**1828**		**889**	**100.0%**	**1.01 [0.89, 1.15]**
Total events	450		264			

Heterogeneity: Tau² = 0.00; Chi² = 0.96, df = 2 (P = 0.62); I² = 0%
Test for overall effect: Z = 0.18 (P = 0.86)

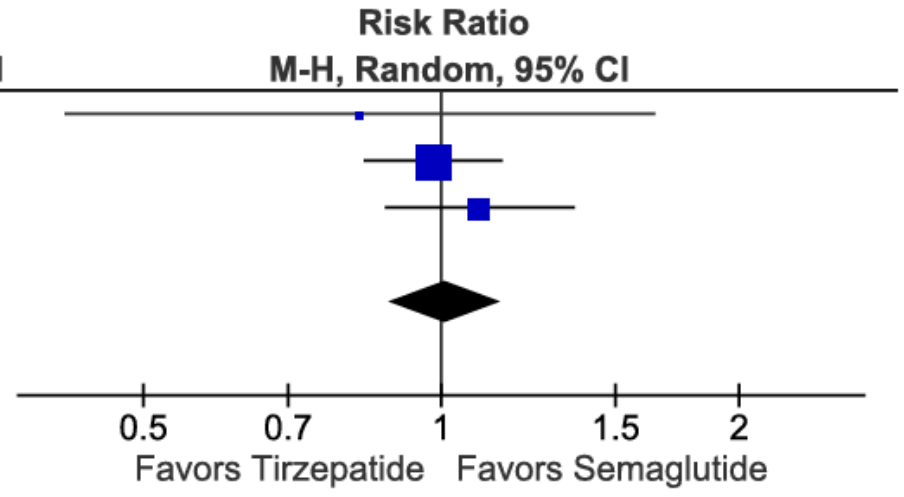

Figure S15: Forest plot of incidence of nausea comparing tirzepatide with semaglutide. No statistically significant difference was observed (RR 1.01; 95% CI 0.89–1.15; p = 0.86; I² = 0%).

Study or Subgroup	Tirzepatide Events	Tirzepatide Total	Semaglutide Events	Semaglutide Total	Weight	Risk Ratio M-H, Random, 95% CI
Heise 2022	3	45	5	44	2.8%	0.59 [0.15, 2.31]
SURMOUNT-5 2025	56	374	80	376	54.3%	0.70 [0.52, 0.96]
SURPASS-2 2021	113	1409	39	469	42.9%	0.96 [0.68, 1.37]
**Total (95% CI)**		**1828**		**889**	**100.0%**	**0.80 [0.64, 1.01]**
Total events	172		124			

Heterogeneity: Tau² = 0.00; Chi² = 1.96, df = 2 (P = 0.38); I² = 0%

Test for overall effect: Z = 1.90 (P = 0.06)

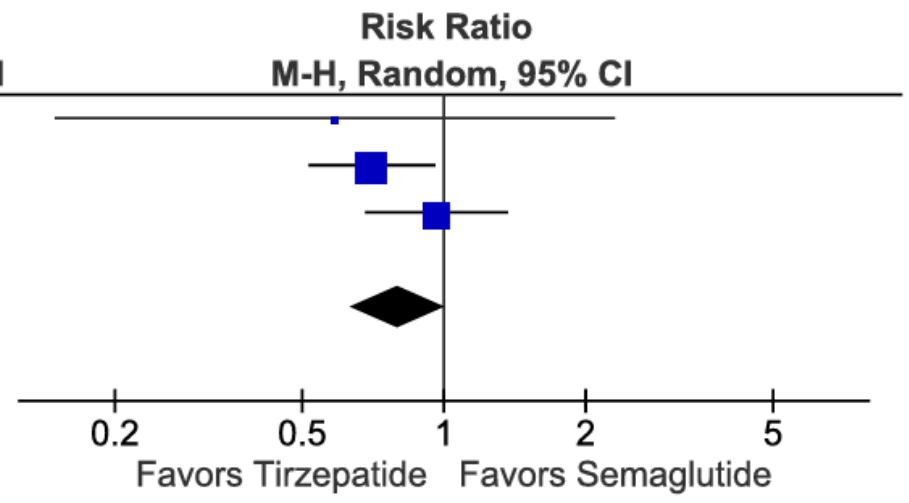

Figure S16: Forest plot of incidence of vomiting comparing tirzepatide with semaglutide. No statistically significant difference was observed (RR 0.80; 95% CI 0.64–1.01; p = 0.06; I² = 0%).

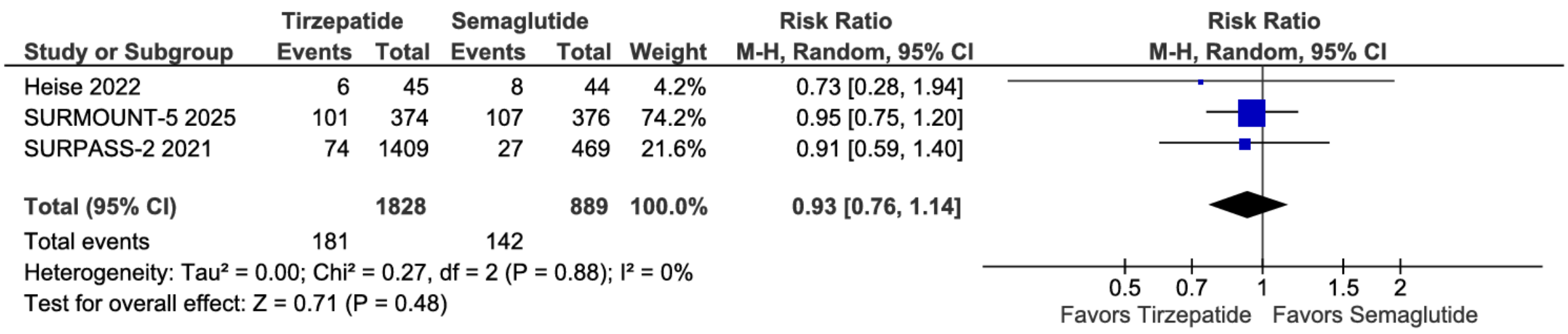

Figure S17: Forest plot of incidence of constipation comparing tirzepatide with semaglutide. No statistically significant difference was observed (RR 0.93; 95% CI 0.76–1.14; p = 0.48; **I²** = 0%).

Study or Subgroup	Tirzepatide Events	Tirzepatide Total	Semaglutide Events	Semaglutide Total	Weight	Risk Ratio M-H, Random, 95% CI
Heise 2022	9	45	13	44	9.8%	0.68 [0.32, 1.42]
SURMOUNT-5 2025	88	374	88	376	47.2%	1.01 [0.78, 1.30]
SURPASS-2 2021	204	1409	54	469	43.0%	1.26 [0.95, 1.67]
**Total (95% CI)**		**1828**		**889**	**100.0%**	**1.07 [0.83, 1.36]**
Total events	301		155			

Heterogeneity: Tau² = 0.02; Chi² = 2.96, df = 2 (P = 0.23); I² = 33%

Test for overall effect: Z = 0.51 (P = 0.61)

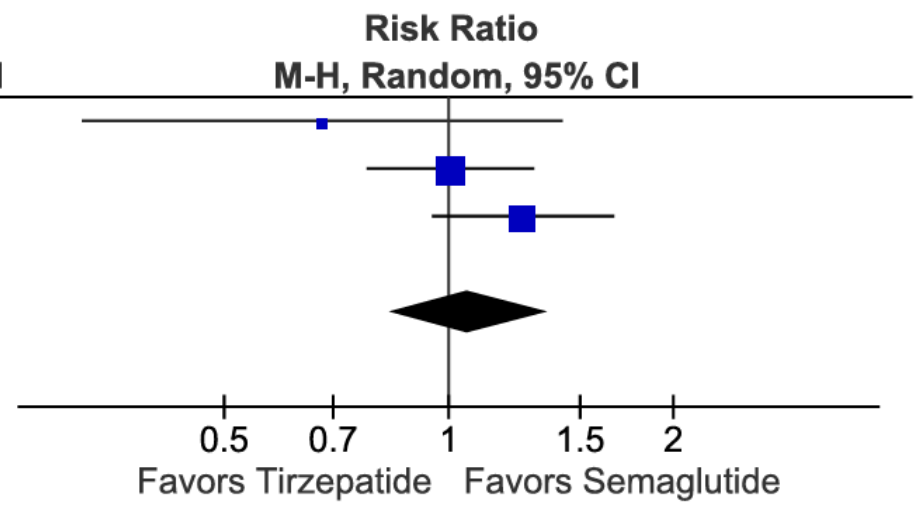

Figure S18: Forest plot of incidence of diarrhea comparing tirzepatide with semaglutide. No statistically significant difference was observed (RR 1.07; 95% CI 0.83–1.36; p = 0.61; I² = 33%).

Study or Subgroup	Tirzepatide Events	Tirzepatide Total	Semaglutide Events	Semaglutide Total	Weight	Risk Ratio M-H, Random, 95% CI
Heise 2022	5	45	4	44	7.3%	1.22 [0.35, 4.25]
SURMOUNT-5 2025	24	374	26	376	39.6%	0.93 [0.54, 1.59]
SURPASS-2 2021	59	1409	24	469	53.1%	0.82 [0.52, 1.30]
**Total (95% CI)**		**1828**		**889**	**100.0%**	**0.89 [0.63, 1.24]**
Total events	88		54			

Heterogeneity: Tau² = 0.00; Chi² = 0.40, df = 2 (P = 0.82); I² = 0%

Test for overall effect: Z = 0.71 (P = 0.48)

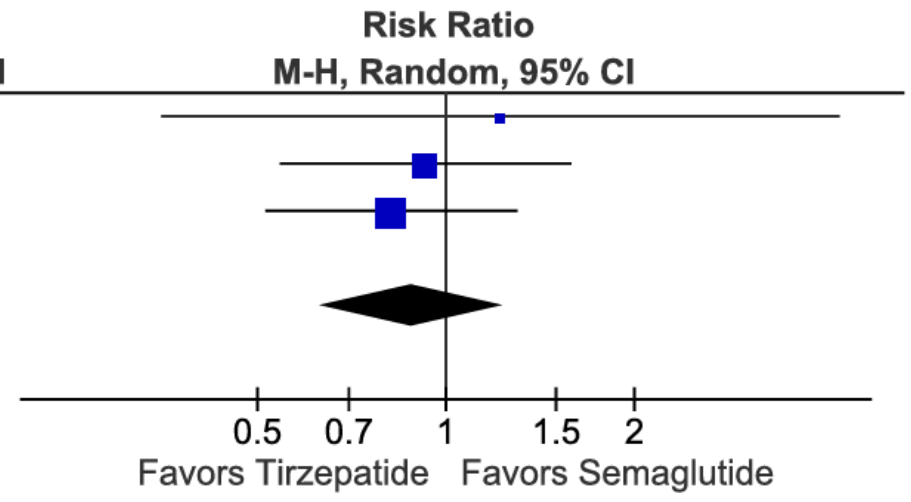

Figure S19: Forest plot of incidence of abdominal pain comparing tirzepatide with semaglutide. No statistically significant difference was observed (RR 0.89; 95% CI 0.63–1.24; p = 0.48; I² = 0%).

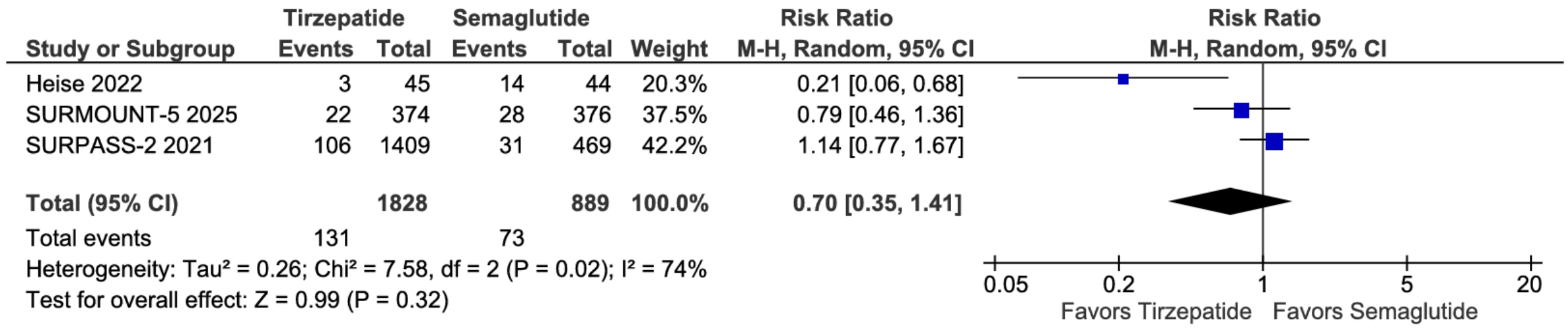

Figure S20: Forest plot of incidence of dyspepsia comparing tirzepatide with semaglutide. No statistically significant difference was observed (RR 0.70; 95% CI 0.35–1.41; $p = 0.32$; $I^2 = 74\%$).

Study or Subgroup	Tirzepatide Events	Tirzepatide Total	Semaglutide Events	Semaglutide Total	Weight	Risk Ratio M-H, Random, 95% CI
Heise 2022	27	45	31	44	41.8%	0.85 [0.63, 1.16]
SURMOUNT-5 2025	17	374	19	376	23.5%	0.90 [0.48, 1.70]
SURPASS-2 2021	111	1409	25	469	34.6%	1.48 [0.97, 2.25]
**Total (95% CI)**		**1828**		**889**	**100.0%**	**1.04 [0.69, 1.58]**
Total events	155		75			

Heterogeneity: Tau² = 0.08; Chi² = 5.33, df = 2 (P = 0.07); I² = 62%

Test for overall effect: Z = 0.21 (P = 0.84)

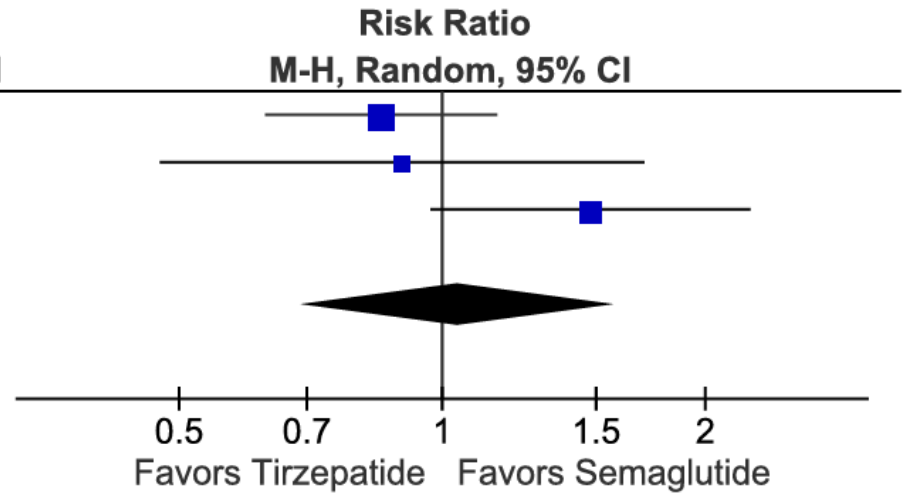

Figure S21: Forest plot of incidence of decreased appetite comparing tirzepatide with semaglutide. No statistically significant difference was observed (RR 1.04; 95% CI 0.69–1.58; p = 0.84; I² = 62%).

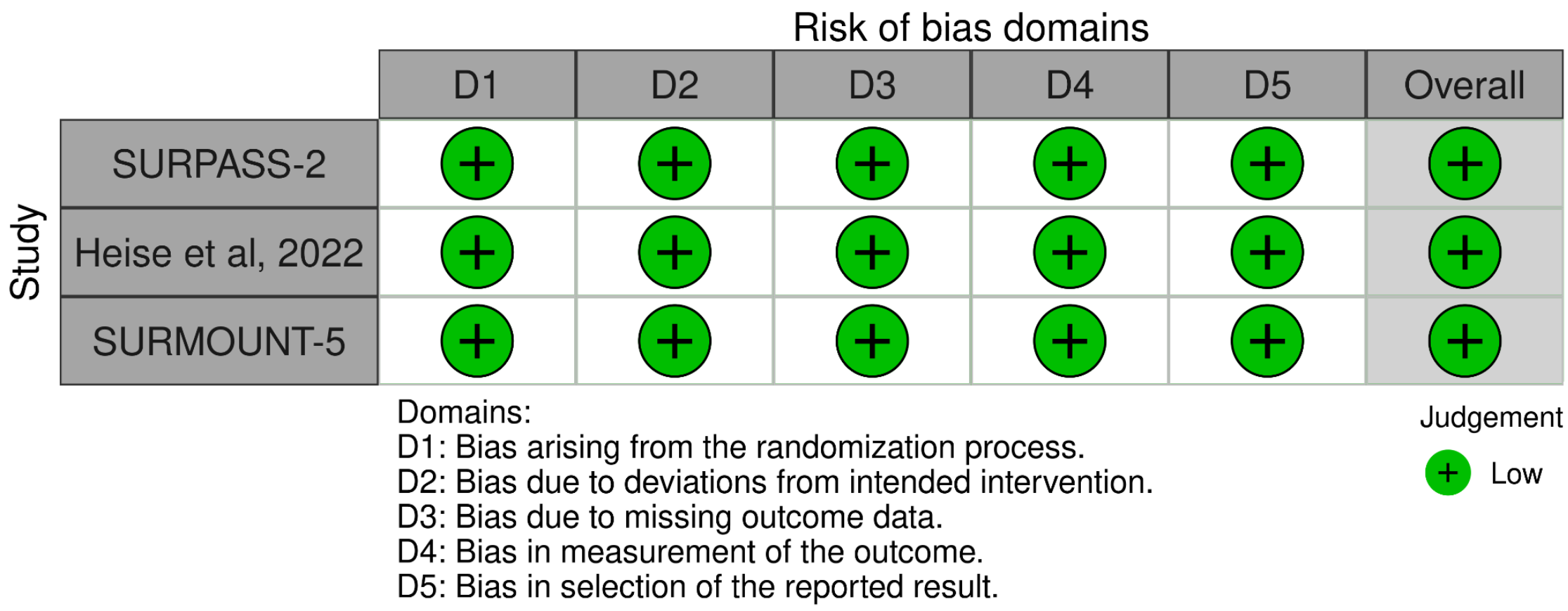

Figure S22: Risk of bias assessment according to the Cochrane Risk of Bias 2 (RoB 2) tool.

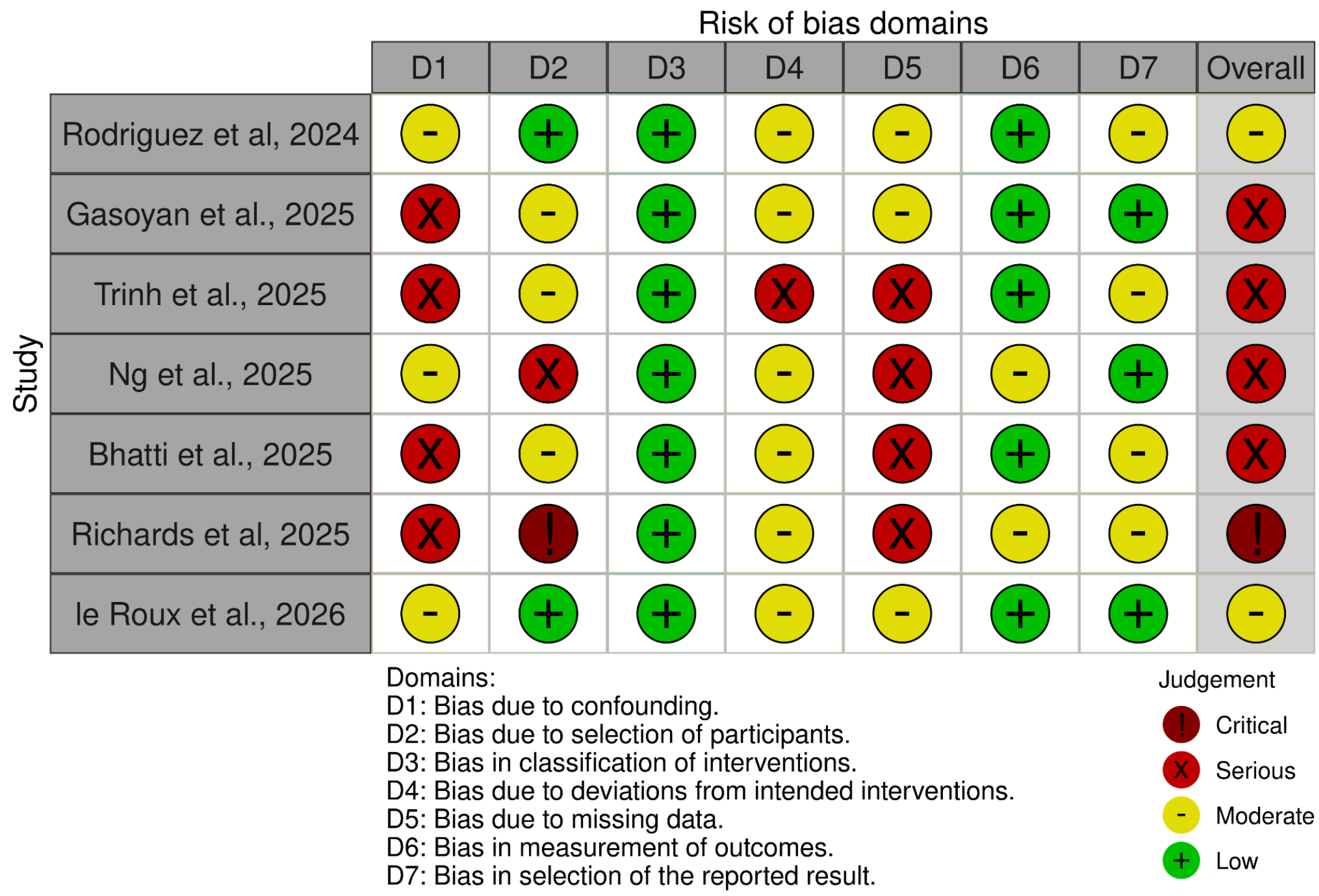

Figure S23: Risk of Bias Assessment Using the ROBINS-I Tool.
